# ProTides of BVdU as potential anticancer agents upon efficient intracellular delivery of their activated metabolites

**DOI:** 10.1016/j.bmcl.2016.10.077

**Published:** 2016-12-01

**Authors:** Sahar Kandil, Jan Balzarini, Stephanie Rat, Andrea Brancale, Andrew D. Westwell, Christopher McGuigan

**Affiliations:** a School of Pharmacy and Pharmaceutical Sciences, Cardiff University, King Edward VII Avenue, Cardiff CF10 3NB, UK; b Rega Institute for Medical Research, KU Leuven, Minderbroedersstraat 10, B-3000 Leuven, Belgium

**Keywords:** BVdU, ProTide, Phosphoramidate, Anticancer, Nucleoside, Prodrug, Brivudine

## Abstract

Nucleosides represent a major chemotherapeutic class for treating cancer, however their limitations in terms of cellular uptake, nucleoside kinase-mediated activation and catabolism are well-documented. The monophosphate pro-nucleotides known as ProTides represents a powerful strategy for bypassing the dependence on active transport and nucleoside kinase-mediated activation. Herein, we report the structural tuning of BVdU ProTides. Forty six phosphoramidates were prepared and biologically evaluated against three different cancer cell lines; murine leukemia (L1210), human CD_4_^+^ T-lymphocyte (CEM) and human cervical carcinoma (HeLa). Twenty-fold potency enhancement compared to BVdU was achieved against L1210 cells. Interestingly, a number of ProTides showed low micromolar activity against CEM and HeLa cells compared to the inactive parent BVdU. The ProTides showed poor, if any measurable toxicity to non-tumourigenic human lung fibroblast cell cultures. Separation of four pairs of the diastereoisomeric mixtures and comparison of their spectral properties, biological activities and enzymatic activation rate is reported.

## Highlights

• Synthesis of 46 BVdU ProTide derivatives with superior activity over BVdU.
• Anti-proliferative activity against L1210, CEM, HeLa and HEL cell lines.
• Up to 20-fold cytostatic activity enhancement over the parent BVdU.
• Retention or increase of the cytostatic activity against TK-deficient cancer cells.
• Separation of 4 pairs of P diastereoisomers and comparison of their properties.

## Graphical abstract

**Figure:**
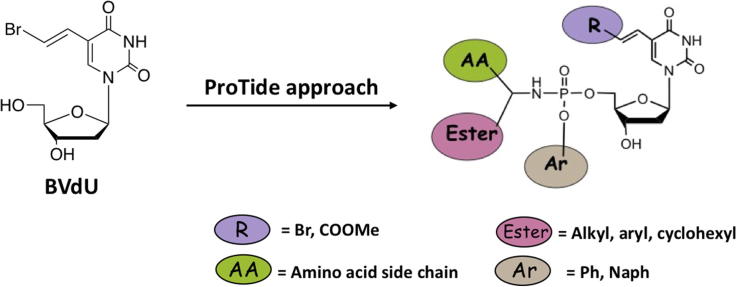

Over the past 40 years, nucleoside analogues have been established as first-line antiviral and anticancer agents. In most cases phosphorylation is required to convert the nucleoside analogues to the corresponding 5’-monophosphates and subsequently to the triphosphates [1], [2], [3]. However, this three-step intracellular conversion is often inefficient in the intact cells being rate-limited by the initial phosphorylation step; furthermore, lower, or lack of expression of nucleoside kinases often leads to emergence of resistance to the nucleoside analogue treatment [1], [2]. Unfortunately, nucleotides cannot be considered as therapeutic agents because of their polar nature, their impermeability through the cell membrane and their dephosphorylation in extracellular fluids. Therefore, considerable efforts have focused on monophosphate prodrugs that carry little or no charge to mask the negative charge of the phosphate group of the nucleotides, with various chemically or enzymatically cleavable moieties once the compound is transported into the cell [2], [3]. Among all reported pro-nucleotide approaches, the class of aryl phosphoramidate nucleosides known as ProTides, pioneered by McGuigan and co-workers, has been proven to enhance the activity of parent nucleosides by improving intracellular transport and/or by bypassing the rate-limiting monophosphorylation step. These improvements serve to eventually increase the formation rate of intracellular nucleoside triphosphate [4]. Proof-of-principle in patients is demonstrated with two FDA-approved therapeutic antiviral agents, Sofosbuvir used for hepatitis C virus treatment and tenofovir alafenamide used for human immunodeficiency virus treatment. Additionally, there are more ProTides in the pipelines of several academic groups and pharmaceutical companies undergoing preclinical and clinical studies for the treatment of viral infections and cancer [2]. The gemcitabine ProTide known as NUC-1031 represents an example of an anticancer ProTide currently in Phase 2 clinical trial [5].

Brivudine, (*E*)-5-(2-bromovinyl)-2′-deoxyuridine, (BVdU) Fig. 1 is a potent inhibitor of herpes simplex virus type 1 (HSV-1) and varicella–zoster virus (VZV). The application of ProTide technology on the BVdU scaffold led to less potent derivatives compared to the parent agent against VZV in cell culture models [6]. This was interpreted as corresponding to poor intracellular delivery of BVdU monophosphate (BVdUMP), rapid degradation to BVdU or poor onward phosphorylation of BVdUMP to the bioactive triphosphate [6]. However, NewBiotics Inc. had independently prepared NB1011 (Thymectacin) which has recently entered Phase I/II clinical trials for the treatment of colon cancer [7], [8]. Further studies on NB1011 revealed that it is selectively toxic to tumour cells expressing elevated levels of thymidylate synthase (TS), a key enzyme in DNA synthesis [9].

Some SAR optimisation of NB1011 was previously reported against human breast (MCF-7, MDA MB 231), prostate (PC3), colon (HT115) and bladder (T24) cancer cell lines [10]. In this work we report the synthesis and biological evaluation of an extensive series of BVdU phosphoramidate derivatives. The tuning of the parent structure involved combined modifications of the amino acid ester, the aromatic masking group on the phosphate moiety and the 5-position of the BVdU nucleoside base. We have synthesized 46 BVdU phosphoramidate derivatives **8**–**53** possessing improved cytotoxic activity against three tumour cell lines; murine leukemia (L1210), human CD_4_^+^ T-lymphocyte (CEM) and human cervical carcinoma (HeLa). Moreover, separation of four phosphorus centre diastereoisomeric pairs was successfully achieved and provided useful comparative insights. Applying the previously reported computational and NMR studies, the absolute stereochemistry of the phosphorus centre of some of these diastereoisomers has been predicted.

BVdU is prepared from 5-iodo-2′-deoxyuridine **1**, which was used to prepare the carboxymethyl ester derivative, **2** via Heck reaction with methyl acrylate in the presence of palladium acetate. Hydrolysis of **2** was carried out using NaOH followed by acidification to get the carboxylic acid derivative **3**, which was treated with *N-*bromosuccinimide (NBS) to give BVdU [11], Scheme 1.

The target BVdU ProTides were all prepared using the extensively described phosphorochloridate chemistry [12], [13]. The phenyl phosphorodichloridate **4a** was used to introduce the phenolic aromatic masking unit of the ProTide. For the naphthyl analogues, 1-naphthol was reacted with phosphoryl chloride to give the required dichloridate **4b**, Scheme 2. The second component of the ProTide motif is an amino acid ester (general formula, **5**), if not commercially available, was prepared by esterification of the appropriate amino acids using standard methods [14]. The arylaminoacyl phosphorochloridate (general formula, **6**), were prepared by reacting the aryl phosphorodichloridate **4a** or **4b** with the amino acid ester **5**, Scheme 2. The formation of the key phosphorochloridate was monitored by ^31^P NMR. Next, the arylaminoacyl phosphorochloridate **6** was reacted with either BVdU or its carboxy methyl ester precursor **2** in the presence of 1-methylimidazole (NMI), Scheme 2. Overnight reactions at ambient temperature generated crude materials that were purified by column chromatography to provide low to moderate yields typical of previous phosphoramidate ProTide syntheses (3.4–28%) [15]. Each of the phosphoramidate compounds was generated as a pair of diastereoisomers at the phosphate center, in roughly 1:1 ratio, as revealed by the two closely spaced peaks in the ^31^P NMR spectrum.

It has been reported before that the biological activity of phosphoramidates can be dependent on the configuration of the phosphorus centre [16], [17]. Single phosphorus centre diastereoisomers were separated from their corresponding racemic mixtures of compounds (**35**, **36**, **47** and **49**) using a combination of gradient column chromatography and preparative thin liquid chromatography. Obtaining four pairs of fast eluting (f) and slow eluting (s) diastereoisomers; (**35**f/**35**s, **36**f/**36**s, **47**f/**47**s and **49**f/**49**s, respectively), offered the opportunity to compare their spectroscopic, anti-proliferative and enzymatic activation rate profiles. Fig. 2A, shows the ^31^P NMR of the diastereoisomeric mixture of **36** and that of its single diastereoisomer components separated (**36**s and **36**f), Fig. 2B and C, respectively. It is noteworthy that in the case of proline-based ProTides (**39** and **51**), we obtained single P diastereoisomers, however the reasons behind this observed stereoselectivity still need to be investigated.

A comparison between the ^1^H NMR spectra of the diastereoisomeric pair (**49**s, **49**f) revealed a characteristic pattern difference of the benzylic methylene protons, Fig. 3. For the **49**f diastereoisomer, the two protons display a singlet signal, Fig. 3A, while the **49**s diastereoisomer shows a double doublet signal, Fig. 3B. Similar findings were reported before for a partially separated racemic mixture of another BVdU ProTide analogue using preparative HPLC and were previously explained through conformational studies [10]. The three aromatic rings (nucleoside base, benzyl ester and naphthyl) are stacked in π-π interactions in the case of the Sp diastereoisomer. This imparts relative rigidity of this conformation and justifies the observed non-equivalent double of doublet splitting NMR pattern of the benzylic methylene hydrogens. On the other hand, the Rp counterpart does not show such interaction among the aromatic rings, resulting in the greater flexibility of the benzylic methylene group reducing the magnetic differences between the two protons and hence, they appear as a singlet signal. Therefore, by combining the NMR and the conformational data, we can propose the Rp configuration to the fast-eluting diastereoisomer **49**f and, consequently, the Sp absolute configuration to the slow-eluting diastereoisomer **49**s.

ProTides **8**–**53** described above were evaluated for their cytostatic activity against a panel of three established tumour cell lines *in vitro*: L1210 (murine leukemia), CEM (human CD_4_^+^ T-lymphocyte), and HeLa (human cervix). In each case a thymidine kinase-deficient (TK^−^) mutant of the parent cell line is included to probe the effect of TK deficiency on the cytostatic activity of the test compounds and the degree to which the ProTides could bypass this dependence. The murine L1210 cell line was included because these tumour cells can be used in a mouse tumour model. Also, a normal human non-tumourigenic primary monolayer cell line (lung fibroblast HEL cells) is included for comparative purposes, and BVdU as a positive parental drug control, Table 1. For the murine leukemia cell line (L1210), alanine-based ProTides were amongst the most active compounds with compound **23** (l-alaninyl benzyl ester naphthoxy phosphoramidate, IC_50_ = 1.8 μM) showing 20-fold increase in potency compared to BVdU (IC_50_ = 38 μM). Interestingly, while BVdU itself does not show any noteworthy anti-proliferative activity against the human T-lymphocyte cell line (CEM) (IC_50_ > 100 μM), most of its phosphoramidate derivatives showed a greatly enhanced activity with compound **37** (l-phenylalaninyl ethyl ester naphthoxy phosphoramidate, IC_50_ = 4.8 μM) being the most active. While BVdU is poorly cytostatic against the human cervical carcinoma (HeLa) (IC_50_ = 160 μM), compounds **33** (l-valinyl cyclohexyl ester naphthoxy phosphoramidate, IC_50_ = 7.2 μM) and **36** (l-tryptophanyl ethyl ester naphthoxy phosphoramidate, IC_50_ = 7.8 μM) were found to boost the antiproliferative activity significantly. Interestingly, BVdU and its phosphoramidate ProTide derivatives generally showed poor, if any cytotoxicity against the human lung fibroblast cell cultures displaying minimal cytotoxic concentration (MCC) values of ⩾100 μM in the vast majority of the test compounds, Table 1. These findings point to a considerable extent of selectivity of the synthesised ProTides, which is potentially beneficial from a drug development viewpoint.

It has been noticed before that BVdU, in contrast to most other thymine-based nucleoside analogues, often shows an increased cytostatic activity against thymidine kinase-deficient tumour cell lines. The molecular/biochemical basis for this phenomenon is still unclear [18]. Here, a pronounced increase of cytostatic activity was also observed for several BVdU ProTides (i.e. **8**, **16**, **21** for L1210 and the majority of compounds for CEM and HeLa). These observations are of particular interest since certain forms of drug resistance of cancer cells have been reported to be caused by thymidine kinase deficiency (i.e. drug resistance against 5-FdUrd- and 5-trifluoromethyl-dUrd-treated cancers) [19], [20]. It would therefore be reasonable to suggest the use of such BVdU ProTides to treat tumours that became refractory to FdUrd/CF_3_dUrd treatment.

All compounds **8**–**53**, were evaluated as mixtures of two phosphate diastereoisomers (Rp and *S*p in 1:1 ratio). Interestingly, we were able to separate four pairs of diastereoisomers, **35**f/**35**s, **36**f/**36**s, **47**f/**47**s and **49**f/**49**s, which offered the opportunity to compare their relative biological activity, Table 1. It could be concluded that in general, the fast eluting diastereoisomer **35**f showed a relatively better cytostatic activity across the three cell lines than its slow eluting diastereoisomer **35**s. Apart from **35**f/**35**s, the other three pairs of diastereoisomers, **36**f/**36**s, **47**f/**47**s and **49**f/**49**s, displayed very similar activity profiles across the three cell lines. In two cases, **36** and **49**, the activity of the parent racemic mixture (1:1 ratio) and that of its single diastereoisomeric components (**36**f/**36**s and **49**f/**49**s, respectively) were evaluated. In both cases the anti-proliferative activity were very similar, Table 1.

The side products from the catabolic conversion of the BVdU ProTides to BVdU-MP are amino acids, and phenol or naphthol. Whereas the release of naturally occurring amino acids should not be a matter of concern in terms of potential side-effects, the release of naphthol or phenol might be. However, it should be noted there are currently two ProTide derivatives approved for clinical use (i.e. TAF, or tenofovir alafenamide [21], [22] and Sofosbuvir [2]) that release phenol upon conversion to the parent compound without measurable toxic side-effects. Also, given the increased activity of some of the BVdU prodrugs against TK-deficient tumour cell lines, the use of BVdU ProTides may perhaps be more efficient in suppression of TK^-^ based resistance development. Generally, the activity profiles suggested that our library of phosphoramidates were able to release the nucleoside monophosphate within intact cells and afford pronounced cytostatic activity in both the wild-type and TK^-^ cancer cell lines, as shown in Table 1.

Nucleoside analogues are challenged by numerous inherent and acquired cancer resistance mechanisms that can significantly limit their effectiveness. ProTides are specifically designed to overcome some key cancer resistance pathways and thereby achieve a superior antineoplastic effect. To exert their anticancer activity, the ProTides are metabolised to release the free nucleoside monophosphate form, which will then generate the corresponding active forms, di- and/or triphosphates. The proposed intracellular activation route of the ProTides has been described for other ProTide families [23] and is exemplified by compound **35** in Scheme 3. The proposed mechanism of activation of the ProTides involves a first enzymatic activation step (i) mediated by a carboxypeptidase-type enzyme that hydrolyses the ester of the aminoacyl moiety to produce the intermediate **A**. Next, a spontaneous cyclization (ii) displacing the aryl moiety via an internal nucleophilic attack of the carboxylate residue on the phosphorus center to yield **B**. In a third step (iii), the unstable ring is hydrolysed to release the intermediate **C**. The last step (iv) involves a phosphoramidase-type enzyme, which cleaves-off the amino acid to generate the corresponding nucleoside monophosphate **D**, Scheme 3. This assay was designed to verify whether an enzymatic cleavage of the ester motif would be sufficient to trigger the first steps of the activation route and generate the intermediate **C** [24].

To probe the difference between the P diastereoisomer activation rate, Scheme 3, an enzymatic study using carboxypeptidase Y while monitoring the conversion by ^31^P NMR was performed. Each diastereoisomer of compound **35**; (**35**f and **35**s) was dissolved in acetone-d_6_ in the presence of Trizma buffer (pH 7.6) and treated with carboxypeptidase Y to monitor the metabolic conversion of the ProTide using ^31^P NMR spectroscopy over time, Fig. 4. In the case of compound **35**f, the experiment showed the hydrolysis of the starting material **35**f (*δ*_P_ = 4.19) to the intermediate type **A** (*δ*_P_ = 5.08), which is then processed to a compound of type **C** (*δ*_P_ = 7.09) through the putative intermediate **B**, Fig. 4A. Interestingly, in the case of compound **35**s, the enzymatic study showed a relatively faster hydrolysis rate of **35**s (*δ*_P_ = 4.13), which is converted directly to compound of type **C** (*δ*_P_ = 7.08), Fig. 4B. These findings demonstrate that phosphoramidate diastereoisomers are processed at different rates by carboxypeptidase-type enzymes.

In an attempt to better understand the difference in the enzymatic activation rate of both diastereoisomers of compound **35** in the enzymatic study, a molecular modelling simulation using the crystal structure of the carboxypeptidase Y (PDB; 1YSC) [25], [26] was performed, Fig. S4 (supporting information, computational study section).

In summary, a series of forty six BVdU ProTides has been synthesised and have been biologically evaluated together with the parent BVdU nucleoside for their cytostatic activity *in vitro* against three different cancer cell lines; murine leukemia (L1210), human T-lymphocyte (CEM) and human cervix carcinoma (HeLa) and for toxicity against human non-tumourigenic lung fibroblast (HEL) cell cultures. ProTide **23** showed twenty-fold better cytostatic activity than the parent BVdU against the L1210 cell line. Low micromolar activity against the CEM and HeLa tumour cell lines compared to inactive parent BVdU nucleoside was observed with a number of our ProTides. The anti-proliferative activity was retained or even enhanced against thymidine kinase-deficient cancer cell lines. Poor, if any cytotoxic activity was observed in HEL cell cultures pointing to a considerable degree of selectivity. Separation of four pairs of P diastereoisomers and the comparison of some spectral properties, anticancer cell activity and enzymatic activation features is described. Molecular modeling studies were conducted in an attempt to explain some of our findings.

Finally, we have demonstrated that the application of the phosphoramidate approach to brivudine (BVdU) results in an extensive source of potential anticancer agents.

## Figures and Tables

**Fig. 1:**
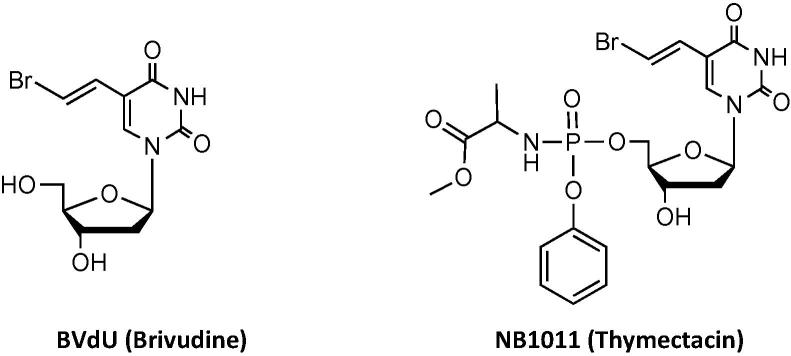
Chemical structure of the BVdU and its ProTide derivative NB1011.

**Fig. 2:**
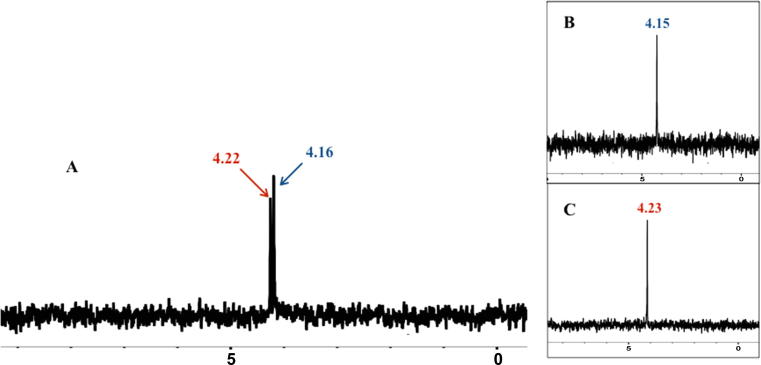
A) ^31^P NMR of the racemic mixture **36**, B) slow eluting diastereoisomer, **36**s, C) fast eluting diastereoisomer **36**f.

**Fig. 3:**
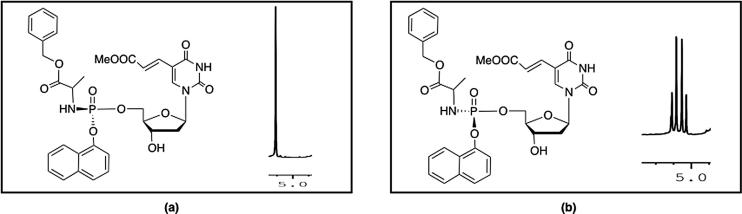
^1^H NMR spectra of the benzylic methylene protons (CH_2_) of the isolated diastereoisomers; (**a**) **49**f, (**b**) **49**s**.**

**Fig. 4:**
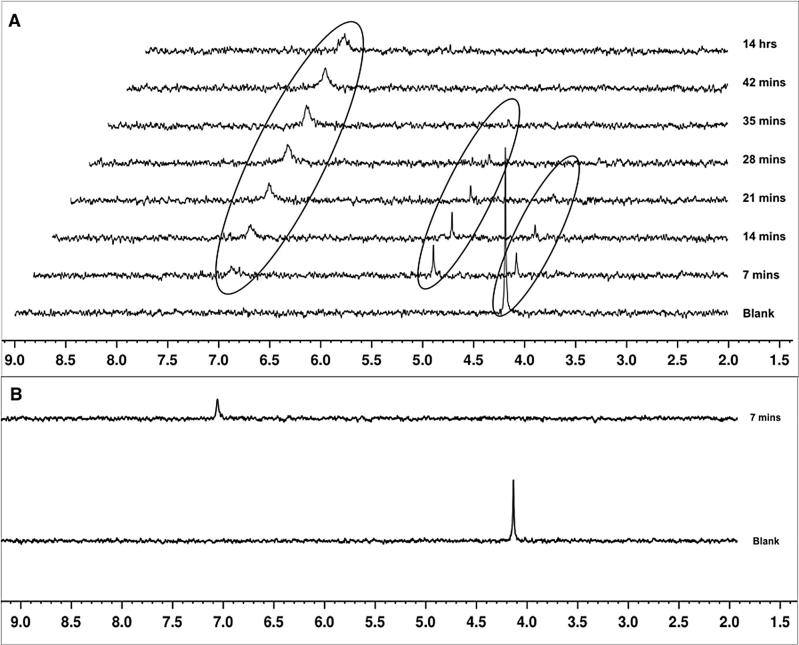
^31^P NMR spectra of ProTide **35**f **(A)** and ProTide **35**s (**B**) over time (every 7 min) after treatment with carboxypeptidase Y (5 mg in acetone-D/Trizma) showing the signals of different metabolites.

**Scheme 1:**
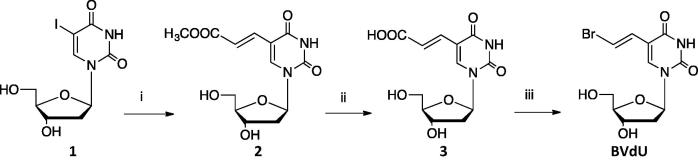
Synthesis of BVdU; Reagents and conditions; i) Pd (OAc)_2_, PPh_3_, Me acrylate, ii) NaOH, HCl, iii) NBS, K_2_CO_3_.

**Scheme 2:**
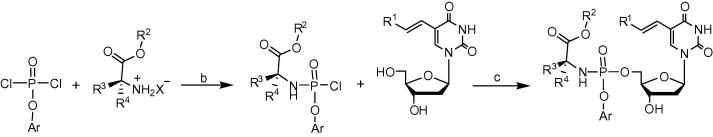
Synthesis of BVdU phosphoramidate analogues; Reagents and conditions: a) Et_3_N, anhydrous DCM, −78 °C, 2–5 h, b) NMI, anhydrous THF, −78 °C to r.t., 16–18 h.

**Scheme 3:**
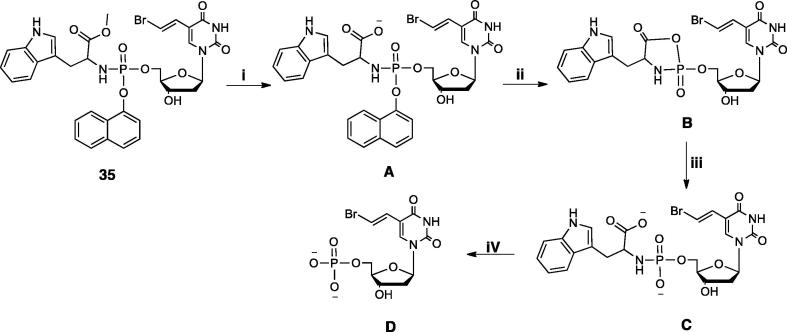
Proposed activation pathway of phosphoramidate **35**; i) esterase or carboxypeptidase-type enzyme, ii) and iii) spontaneous, iv) phosphoramidase-type enzyme.

**Table 1:** Inhibitory effects of BVdU ProTides on the proliferation of murine leukemia (L1210, L1210/TK^-^), human CD_4_^+^ T-lymphocyte (CEM, CEM/TK^-^) and human cervical carcinoma (HeLa, HeLa/TK^−^) cells, and their toxicity against human non-tumourigenic lung fibroblast (HEL) cultures.

No.	R	Ar	Ester	AA	IC_50_ (μM)						MCC^a^
					L1210/0	L1210/TK^−^	Cem/0	Cem/TK^−^	HeLa	HeLa/TK^−^	HEL
**8**	Br	Ph	Bn	Ala	2.8 ± 0.3	0.35 ± 0.1	36 ± 1.0	32 ± 2.0	38 ± 2.0	3.1 ± 1.7	⩾100
**9**	Br	Ph	Et	Ala	17 ± 5.0	2.0 ± 1.4	32 ± 11.0	21 ± 0.0	48 ± 7.0	9.6 ± 1.8	100
**10**	Br	Ph	Me	Trp	13.2 ± 0.3	6.7 ± 0.6	24 ± 0.6	16 ± 1.0	33 ± 6.0	1.2 ± 0.0	100
**11**	Br	Ph	Et	Trp	18 ± 1.0	4.2 ± 0.2	14 ± 6.0	14 ± 2.0	18 ± 3.0	2.7 ± 0.1	100
**12**	Br	Ph	Me	Phe	32 ± 2.0	6.2 ± 0.7	23 ± 2.0	6.7 ± 3.2	34 ± 2.0	2.8 ± 1.8	>100
**13**	Br	Ph	Et	Phe	30 ± 3.0	5.6 ± 0.7	23 ± 4.0	9.5 ± 2.3	40 ± 4.0	3.8 ± 2.8	>100
**14**	Br	Ph	cHex	Val	9.3 ± 0.2	10 ± 0.0	8.6 ± 1.1	10 ± 0.0	12 ± 0.0	7.4 ± 0.4	>100
**15**	Br	Ph	Et	Tyr	48 ± 5.0	26 ± 4.0	>100	>100	47 ± 9.0	8.2 ± 2.2	>100
**16**	Br	Ph	cHex	Ala	2.6 ± 0.2	1.5 ± 0.0	15 ± 4.0	25 ± 5.0	16 ± 0.0	6.5 ± 1.7	>100
**17**	COOMe	Ph	Me	Ala	14 ± 0.0	15 ± 2.0	34 ± 6.0	47 ± 1.0	10 ± 0.0	6.1 ± 1.0	>100
**18**	COOMe	Ph	Et	Phe	67 ± 13.0	57 ± 6.0	55 ± 2.0	24 ± 11.0	80 ± 10.0	35 ± 1.0	>100
**19**	COOMe	Ph	Me	Phe	>100	>100	>100	69 ± 43.0	>100	66 ± 31.0	>100
**20**	COOMe	Ph	cHex	Val	75 ± 8.0	79 ± 3.0	69 ± 8.0	81 ± 7.0	⩾ 100	38 ± 7.0	>100
**21**	COOMe	Ph	Bn	Ala	4.0 ± 2.3	36 ± 5.0	19 ± 2.0	51 ± 6.0	32 ± 5.0	42 ± 9.0	>100
**22**	Br	Nap	NeoPnt	Ala	15 ± 2.0	0.89 ± 0.08	13 ± 1.0	13 ± 4.0	32 ± 13.0	4.7 ± 1.3	100
**23**	Br	Nap	Bn	Ala	1.8 ± 0.0	0.24 ± 0.3	9.3 ± 3.9	5.4 ± 0.9	33 ± 17.0	1.1 ± 0.3	⩾100
**24**	Br	Nap	cHex	Ala	9.2 ± 3.7	2.9 ± 0.0	6.1 ± 0.2	5.8 ± 0.6	32 ± 20.0	3.0 ± 1.6	20
**25**	Br	Nap	Et	Ala	8.0 ± 5.2	0.47 ± 0.2	28 ± 12.0	16 ± 3.0	84 ± 13.0	4.7 ± 0.0	>100
**26**	Br	Nap	iPr	Ala	18 ± 1.0	1.6 ± 0.2	14 ± 4.0	15 ± 1.0	21 ± 1.0	5.5 ± 0.3	100
**27**	Br	Nap	Bn	Met	⩾ 250	167 ± 69.0	>250	>250	>250	22.0 ± 9.0	⩾20
**28**	Br	Nap	Bn	Val	7.1 ± 2.3	10 ± 4.0	6.0 ± 0.1	5.2 ± 0.3	12 ± 5.0	3.6 ± 0.3	10
**29**	Br	Nap	Bn	Pro	15 ± 1.0	16 ± 1.0	9.9 ± 0.9	12 ± 0.0	23 ± 2.0	5.2 ± 3.5	⩾4
**30**	Br	Nap	2-Bu	Ala	17 ± 2.0	3.1 ± 0.5	15 ± 2.0	15 ± 3.0	23 ± 1.0	4.3 ± 2.4	⩾100
**31**	Br	Nap	Bn	Gly	18 ± 3.0	3.9 ± 0.1	18 ± 2.0	19 ± 1.0	36 ± 19.0	1.1 ± 0.6	100
**32**	Br	Nap	Bn	d-Ala	21 ± 3.0	19 ± 1.0	17 ± 0.0	18 ± 3.0	31 ± 8.0	7.7 ± 5.0	⩾100
**33**	Br	Nap	cHex	Val	8.3 ± 0.2	8.8 ± 0.1	7.4 ± 1.3	8.4 ± 1.5	7.2 ± 0.2	5.0 ± 0.9	⩾100
**34**	Br	Nap	Et	Val	16 ± 3.0	16 ± 3.0	8.4 ± 1.8	9.2 ± 2.3	27 ± 5.0	7.2 ± 1.1	⩾100
**35f**	Br	Nap	Me	Trp	7.7 ± 0.9	2.4 ± 0.3	9.6 ± 1.7	8.0 ± 0.4	26 ± 14.0	3.2 ± 1.9	100
**35s**					40 ± 2.0	28 ± 9.0	39 ± 5.0	26 ± 2.0	43 ± 3.0	17 ± 7.0	100
**36**					8.3 ± 0.6	4.0 ± 0.9	7.1 ± 0.5	6.1 ± 1.1	7.8 ± 0.7	6.0 ± 1.4	–
**36 f**	Br	Nap	Et	Trp	8.7 ± 0.7	2.5 ± 0.1	9.8 ± 1.5	7.6 ± 1.6	3.9 ± 1.3	2.3 ± 1.2	⩾100
**36s**					8.2 ± 0.3	7.0 ± 0.5	7.8 ± 0.6	5.8 ± 1.3	8.2 ± 0.1	3.1 ± 0.4	35
**37**	Br	Nap	Et	Phe	7.5 ± 0.4	8.2 ± 0.1	4.8 ± 1.0	4.6 ± 1.0	18 ± 3.0	2.8 ± 0.4	100
**38**	Br	Nap	Me	Phe	18 ± 1.0	14 ± 0.0	13 ± 8.0	6.3 ± 1.1	73 ± 0.0	1.8 ± 0.8	100
**39**	Br	Nap	Et	Pro	27 ± 7.0	14 ± 6.0	11 ± 1.0	9.5 ± 1.4	30 ± 3.0	6.4 ± 1.0	>100
**40**	Br	Nap	Et	OMeTyr	19 ± 8.0	6.8 ± 2.1	13 ± 1.0	6.6 ± 2.8	32 ± 2.0	6.1 ± 1.2	100
**41**	Br	Nap	*n-*Pnt	Val	23 ± 5.0	12 ± 3.0	29 ± 1.0	12 ± 2.0	33 ± 6.0	10 ± 0.0	>100
**42**	Br	Nap	*n-*Pnt	Phe	13 ± 1.0	9.6 ± 0.4	17 ± 2.0	10 ± 0.0	38 ± 7.0	9.3 ± 0.0	100
**43**	Br	Nap	Et	Tyr	8.4 ± 0.3	6.9 ± 0.4	9.6 ± 0.1	8.2 ± 0.5	38 ± 1.0	1.7 ± 0.8	>100
**44**	Br	Nap	Et	Met	26 ± 0.0	8.3 ± 0.0	19 ± 9.0	12 ± 4.0	36 ± 0.0	11 ± 3.0	>100
**45**	Br	Nap	cHex	Gly	15 ± 5.0	6.9 ± 0.5	11 ± 4.0	9.5 ± 0.2	29 ± 10.0	6.3 ± 2.1	100
**46**	Br	Nap	Neo-pnt	DMG	9.2 ± 1.2	3.0 ± 1.7	10 ± 2.0	8.6 ± 0.0	25 ± 6.0	7.4 ± 0.3	⩾20
**47f**	COOMe	Nap	Et	Trp	8.8 ± 0.0	8.9 ± 0.9	11 ± 0.0	6.2 ± 2.3	36 ± 4.0	19 ± 13.0	>100
**47s**					11 ± 1.0	14 ± 0.0	11 ± 0.0	8.9 ± 0.8	34 ± 1.0	32 ± 7.0	100
**48**	COOMe	Nap	Et	Val	56 ± 2.0	40 ± 3.0	49 ± 3.0	47 ± 1.0	79 ± 7.0	15 ± 10.0	>100
**49**					42 ± 5.0	32 ± 2.0	35 ± 8.0	46 ± 4.0	57 ± 0.0	26 ± 10.0	>100
**49f**	COOMe	Nap	Bn	Ala	32 ± 6.0	22 ± 9.0	27 ± 2.0	20 ± 10.0	37 ± 1.0	29 ± 9.0	100
**49s**					35 ± 6.0	21 ± 8.0	29 ± 3.0	32 ± 8.0	37 ± 4.0	11 ± 6.0	100
**50**	COOMe	Nap	cHex	Val	16 ± 1.0	16 ± 7.0	10 ± 1.0	8.1 ± 1.7	29 ± 2.0	11 ± 2.0	>100
**51**	COOMe	Nap	Et	Pro	54 ± 4.0	46 ± 3.0	38 ± 2.0	42 ± 1.0	⩾ 100	40 ± 2.0	>100
**52**	COOMe	Nap	*n-*Pnt	Phe	10 ± 0.0	12 ± 1.0	12 ± 3.0	8.1 ± 1.5	33 ± 6.0	9.7 ± 0.2	>100
**53**	COOMe	Nap	Et	Met	26 ± 3.0	50 ± 7.0	52 ± 8.0	58 ± 8.0	62 ± 11.0	35 ± 9.0	>100
		**BVdU**			**38** ± **4.0**	**9.6** ± **2.8**	>**100**	**100**	**160** ± **21.0**	**0.20** ± **0.16**	**–**

a MCC or minimal cytotoxic concentration in (μM) required to afford a microscopically visible alteration of cell morphology.

## A Supplementary data

Supplementary data 1 Experimental procedures and spectroscopic characterisation data of the compounds **8**–**53** as well as the computational study.

### MOL files

The following ZIP file contains the MOL files of the most important compounds referred to in this article.

MOL files ZIP file containing the MOL files of the most important compounds in this article.
