# Supplementary material for: ProTides of BVdU as potential anticancer agents upon efficient intracellular delivery of their activated metabolites
Source: Bioorg Med Chem Lett. 2016 Dec 1;26(23):5618–23. doi: 10.1016/j.bmcl.2016.10.077 (PMC5131913; doi:10.1016/j.bmcl.2016.10.077)
Supplement: Supplementary data 1 — Experimental procedures and spectroscopic characterisation data of the compounds 8–53 as well as the computational study. [file mmc1.docx]

**Supporting Information**

**ProTides of BVdU as Potential Anticancer Agents**

Sahar Kandil^a^^[[1]](#footnote-1)^, Stephanie Rat^a^, Jan Balzarini^b^, Andrea Brancale^a^, Andrew D. Westwell^a^ and Christopher McGuigan^a^

*^a^School of Pharmacy and Pharmaceutical Sciences, Cardiff University, King Edward VII Avenue, Cardiff CF10 3NB, U.K.*

*^b^Rega Institute for Medical Research, KU Leuven, Minderbroedersstraat 10, B-3000 Leuven, Belgium.*

***“This work is dedicated to the memory of Professor Christopher McGuigan”***

**Contents**

S1 Chemistry.

S2 Cytostatic Activity Assays

S3 Enzymatic Procedure: Carboxypeptidase Y assay

S4 Computational study

**S1 Chemistry**

The anhydrous solvents and any other used commercially available reagents were bought from Sigma-Aldrich and used without further purification. Amino acid esters were purchased from Carbosynth. Carboxypeptidase Y and buffers were from Sigma-Aldrich. All reactions were carried out under an argon atmosphere. Reactions were monitored with analytical TLC on silica gel 60-F254 precoated aluminum plates and visualized under UV (254 nm) and/or with ^31^P NMR spectra. Column chromatography was performed on silica gel (35-70 μM). Preparative TLC plates (20 cm × 20 cm, 500-2000 μm) were purchased from Merck. Proton (^1^H), carbon (^13^C) and phosphorus (^31^P) NMR spectra were recorded on a Bruker Avance 500 spectrometer at 25 °C. Spectra were autocalibrated to the deuterated solvent peak, and all ^13^C NMR and ^31^P NMR were proton-decoupled. The purity of the final compounds was verified to be >95% by HPLC analysis using either I) ThermoSCIENTIFIC, SPECTRA SYSTEM P4000, detector SPECTRA SYSTEM UV2000, Varian Pursuit XRs 5 C18, 150 x 4.6 mm (as an analytic column) or II) Varian Prostar (LC Workstation-Varian Prostar 335 LC detector), Thermo SCIENTIFIC Hypersil Gold C18, 5μ, 150 x 4.6 mm (as an analytic column) with a gradient elution of H_2_O/ CH_3_CN from 100/0 to 0/100 in 35 min, Flow = 1 mL/min, λ = 275 nm. Mass spectra were performed on Bruker Daltonics microTof-LC, (atmospheric pressure ionization, electron spray mass spectroscopy) in either positive or negative mode. Mass spectroscopy was performed as a service by School of Chemistry at Cardiff University, using fast atom bombardment (FAB).

**General experimental procedure for the preparation of BVdU ProTides (8-53);**

BVdU phosphoramidates (**8**-**53**) were prepared applying the extensively described phosphochloridate chemistry.^12,13^ To a Stirring of solution of (E)-5-(2-bromovinyl)-2’-deoxyuridine, BVdU or 5-(2-carboxymethoxyvinyl)-2’-deoxyuridine, **2**, (1.0mol eq.) and the appropriate phosphochloridate (3.0 mol eq.) in anhydrous THF at –78 **°**C; NMI (5.0 mol eq.) was added dropwise over 1 min. After 30 min the reaction was left to rise to room temperature and stirred for 12 h. The solvent was removed under reduced pressure and the yellow oil obtained was dissolved in DCM, washed with 0.5 M HCl and water. The organic layer was dried over MgSO_4_, filtered, reduced to dryness and purified by flash chromatography.

**(E)-5-(2-bromovinyl)-2’-deoxyuridine-5’-(1-phenyl(benzyloxy-L-alaninyl) phosphate (8)**

Purified by column chromatography (CH_2_Cl_2_/MeOH from 100/0 to 97/3), white solid, yield 21.9%.

^31^P NMR (202MHz, MeOD) δ 4.31, 3.84. ^1^H-NMR (500 MHz; MeOD) δ 7.78-7.71 (m, 1H, *H*-6), 7.42-7.29 (m, 8H, Ar*H, H*-5b), 7.28-7.16 (m, 3H, Ar*H*), 6.87-6.78 (m, 1H, *H*-5a), 6.29-6.19 (m, 1H, *H*-1’), 5.18-5.12 (m, 2H, C*H*_2_Ph), 4.47-4.31 (m, 3H, *H*-3’, *H*-5’), 4.13-4.07 (m, 2H, CH_3_C*H*, *H*-4’), 2.34-2.24 (m, 1H, *H*-2’), 2.13-2.03 (m, 1H, *H*-2’), 1.39-1.31 (m, 3H, C*H*_3_). ^13^C-NMR (125 MHz; MeOD) δ 174.73, 174.70 (C=O ester), 163.64 (*C-*4), 151.14 (*C-*2), 140.01 (*C-*6), 147.01, 137.38, 130.91, 130.53, 129.79, 129.36, 126.41, 121.56, 116.41 (*C*-5a, Ph*,* *C*-5), 109.36 (*C*-5b), 87.26, 86.98 *(C*-1’), 86.30 (*C*-4’), 72.21 *(C*-3’), 71.77 (Ph*C*H_2_ ester), 67.88 (*C*-5’), 51.85 (CH_3_*C*H), 41.40 *(C*-2’), 20.60, 20.49 (*C*H_3_). MS [ESI, m/z]: 672.06 [M+Na]. t_R_ = 13.48 min.

**(E)-5-(2-bromovinyl)-2’-deoxyuridine-5’-[phenyl(ethyloxy-L-alaninyl)]phosphate (9)**

Purified by column chromatography (CH_2_Cl_2_/MeOH from 100/0 to 97/3), white solid, yield 14 %. ^31^P-NMR (202MHz, CDCl_3_), δ 3.60, 3.21. ^1^H-NMR (500MHz, CDCl_3_), δ 9.32 (bs, 1H, O*H*-3), 7.61 (d, *J* = 8.1 Hz, 1H, *H*-6), 7.531 (m, 1H, *H*-5b), 7.47-7.24 (m, 5H, Ar*H*), 6.79 (m, 1H, *H*-5a), 6.22 (m, 1H, *H*-1’), 4.51-4.30 (m, 4H, *H*-3’, *H*-4’, *H*-5’), 4.18 (m, 2H, OC*H*_2_CH_3_), 4.17-3.91 (m, 2H, m, CH_3_C*H*, N*H*), 2.41 (m, 1H, *H*-2’), 2.14-2.12 (m, 1H, *H*-2’), 1.41 (m, 3H, C*H*_3_CH), 1.31 (m, 3H, OCH_2_C*H*_3_); ^13^C-NMR (125MHz, CDCl_3_) δ 174.61 (*C*=O), 163.44 (*C*-2), 151.23 (*C*-4), 147.08 (*ipso* Ph), 139.76 (*C*-6), 120.07, 126.41, 129.89 (*C*-2 Ph, *C*-3 Ph, *C*-4 Ph, *C*-5 Ph, *C*-6 Ph, *C*-5a), 111.52 (*C-*5), 109.46 (*C*-5b), 86.90, 87.06 (*C*-1’), 86.82 (*C*-4’), 72.14, 72.18 (*C*-3’), 67.78 (*C*-5’), 61.86 (O*C*H_2_CH_3_), 50.35, 50.47 (*C*HCH_3_), 41.06, 41.37 (*C*-2’), 20.85 (CH*C*H_3_), 14.11 (OCH_2_*C*H_3_). MS [ESI, m/z]: 609.96 [M+Na]. t_R_ = 12.92min.

**(E)-5-(2-bromovinyl)-2’-deoxyuridine-*5’-*(1-phenyl(methoxy-L-tryptophanyl)phosphate (10)**

Purified by column chromatography (CH_2_Cl_2_/MeOH from 100/0 to 97/3), white solid, yield 15.8 %. ^31^P NMR (202MHz, MeOD) δ_P_ 4.01, 3.87. ^1^H-NMR (500 MHz; MeOD) δ 7.63-7.60 (m, 1H, Ar*H*), 7.53-7.50 (m, 1H, Ar*H*), 7.39-7.21 (m, 3H, *H*-6, *H*-5b, Ar*H*), 7.19-7.00 (m, 7H, Ar*H)*, 6.78-6.72 (m, 1H, *H*-5a), 6.21-6.15 (m, 1H, *H-*1’), 4.30-4.20 (m, 1H, *H*-3’), 4.19-4.11 (m, 1H, C*H*CH_2_-Trp), 4.07-3.93 (m, 3H, *H*-5’, *H*-4’), 3.68-3.09 (m, 3H, C*H*_3_), 3.30-3.22 (m, 1H, CHC*H*_2_-Trp), 3.13-3.05 (m, 1H, CHC*H*_2_-Trp), 2.29-2.20 (m, 1H, *H*-2’), 2.00-1.92 (m, 1H, *H*-2’).^13^C-NMR (125 MHz; MeOD), δ 174.63 (C=O ester), 163.57 (*C-*4), 151.21 (*C-*2), 139.67 (*C-*6), 138.18, 131.24, 130.66, 130.51, 130.49, 128.75, 128.71, 126.23, 126.19, 124.81, 124.79, 122.52, 121.49, 121.45, 121.31, 121.25, 119.83, 119.27, 112.71, 112.39, 112.26, 111.03, (*C*-5, ipso Ph*, C*-2 Ph, *C*-3 Ph, *C*-4 Ph, *C*-5 Ph, *C*-6 Ph, *C*-2 Trp, *C*-3 Trp, *C*-3a Trp, *C*-4 Trp, *C*-5 Trp, *C*-6 Trp, *C*-7 Trp, *C*-7a Trp, *C*-5a), 109.29 (*C*-5b), 87.12, 86.99 (*C*-4’), 86.73, 86.67 *(C*-1’), 72.19, 72.15 *(C*-3’), 67.44 (*C*-5’) , 57.36, 57.27 (CH_2_*C*H Trp), 52.45, 52.39 (*C*H_3_), 41.35, 41.28 *(C*-2’), 31.13 (d, ^3^*J*_C-P_= 8.75 Hz, *C*H_2_CH Trp). MS [ESI, m/z]: 711.19 [M+Na], t_R_ = 12.67 min.

**(E)-5-(2-bromovinyl)-2’-deoxyuridine*-5’-*(1-phenyl(ethoxy-L-tryptophanyl)-phosphate (11)**

Purified by column chromatography (CH_2_Cl_2_/MeOH from 100/0 to 97/3), white solid, yield 11.4%.

^31^P NMR (202MHz, MeOD) δ 4.06, 3.94. ^1^H-NMR (500MHz; MeOD) δ 7.68-7.61 (m, 1H, Ar*H*), 7.58-7.51 (m, 1H, Ar*H*), 7.40-7.21 (m, 4H, *H*-6, *H*-5b, Ar*H*), 7.21-7.00 (m, 6H, Ar*H)*, 6.81-6.72 (2d, *J* = 13.6 Hz, 1H, *H*-5a), 6.23-6.15 (m, 1H, *H-*1’), 4.33-4.22 (m, 1H, *H*-3’), 4.18-4.11 (m, 1H, C*H*CH_2_-Trp), 4.10-3.87 (m, 5H, *H*-5’, C*H_2_*CH_3_, *H*-4’), 3.31-3.22 (m, 1H, CHC*H*_2_-Trp), 3.12-3.05 (m, 1H, CHC*H*_2_-Trp), 2.29-2.19 (m, 1H, *H*-2’), 1.99-1.91 (m, 1H, *H*-2’), 1.18-1.10 (m, 3H, C*H*_3_). ^13^C-NMR (125 MHz; MeOD) δ 174.67 (C=O ester), 163.61 (*C-*4), 151.00 (*C-*2), 139.61 (*C-*6), 138.08, 130.78, 130.75, 130.53, 130.51, 128.77, 128.65, 126.25, 126.14, 124.92, 124.84, 122.53, 121.52, 121.48, 121.24, 121.20, 119.93, 119.23, 112.42, 112.37, 112.27, 110.76, (*C*-5, ipso Ph*, C*-2 Ph, *C*-3 Ph, *C*-4 Ph, *C*-5 Ph, *C*-6 Ph, *C*-2 Trp, *C*-3 Trp, *C*-3a Trp, *C*-4 Trp, *C*-5 Trp, *C*-6 Trp, *C*-7 Trp, *C*-7a Trp, *C*-5a), 109.27 (*C*-5b), 87.06, 86.94 (*C*-4’), 86.75, 86.69 *(C*-1’), 72.17, 72.13 *(C*-3’), 67.41 (*C*-5’), 62.43, 62.36 (*C*H_2_ ester), 57.34, 57.25 (CH_2_*C*H Trp), 41.34, 41.26 *(C*-2’), 31.06 (d, ^3^*J*_C-P_= 8.75 Hz, *C*H_2_CH Trp), 14.38 (*C*H_3_). MS [ESI, m/z]: 725.19 [M+Na], t_R_ = 13.37 min.

**(E)-5-(2-bromovinyl)-2’-deoxyuridine-***5’-***(1-phenyl(methoxy-L-phenylalanyl)-phosphate (12)**

Purified by column chromatography (CH_2_Cl_2_/MeOH from 100/0 to 97/3), white solid, yield 20 %.

^31^P NMR (202 MHz, MeOD) δ 3.98, 3.77. ^1^H-NMR (500 MHz, MeOD) δ 7.71 (s, 1H, *H*-6)*,* 7.42- 7.38 (m, 1H, *H*-5b), 7.33-7.08 (m, 10H, Ar*H)*, 6.82-6.78 (m, 1H, *H*-5a), 6.29-6.19 (m, 1H, *H-*1’), 4.41-4.29 (m, 1H, *H*-3’), 4.22-4.10 (m, 2H, *H*-4’, C*H*CH_3_), 4.09-4.00 (m, 2H, *H*-5’), 3.18, 3.12 (2 s, 3H, CH_3_), 3.13-3.09 (m, 1H, CHC*H*_2_), 2.92-2.88 (m, 1H, CHC*H*_2_), 2.31-2.25 (m, 1H, *H*-2’), 2.11-1.99 (m, 1H, *H*-2’).^13^C-NMR (125MHz; MeOD), δ 174.21 (C=O ester), 163.60 (*C-*4), 151.04 (*C-*2), 139.68 (*C-*6), 139.68, 138.20, 130.81, 130.58, 130.56, 130.53, 129.64, 129.61, 127.99, 126.29, 126.15, 121.60, 121.56, 121.25, 121.21, 112.32, 112.28, (*C*-5, *Ar* Phe, *Ar* Ph, *C*-5a), 109.33, 109.29 (*C*-5b), 87.13, 86.98 (*C*-4’), 86.77, 86.73 *(C*-1’), 72.23, 72.13 *(C*-3’), 67.47, 67.44, 67.40 (*C*-5’), 57.93, 57.83 (CH_2_*C*H Phe), 52.76, 52.71 (*C*H_3_), 41.40, 41.25, 41.00 *(C*-2’), 40.94, 40.90, 40.83 (*C*H_2_CH). MS [ESI, m/z]: 650.08 [M+H]. t_R_ = 13.76 min

**(E)-5-(2-bromovinyl)-2’-deoxyuridine-***5’-***(1-phenyl(ethoxy-L-phenylalaninyl)-phosphate (13)**

Purified by column chromatography (CH_2_Cl_2_/MeOH from 100/0 to 97/3), white solid, yield 21.4 %. ^31^P NMR (202 MHz, MeOD) δ 3.97, 3.79. ^1^H-NMR (500 MHz, MeOD) δ 7.70 (s, 1H, *H*-6)*,* 7.43- 7.39 (m, 1H, *H*-5b), 7.32-7.05 (m, 10H, Ar*H)*, 6.81-6.77 (m, 1H, *H*-5a), 6.30-6.20 (m, 1H, *H-*1’), 4.43-4.31 (m, 1H, *H*-3’), 4.22-4.00 (m, 6H, *H*-4’, C*H*CH_2_, C*H_2_*CH_3_, *H*-5’), 3.12-3.05 (m, 1H, CHC*H*_2_), 2.93-2.88 (m, 1H, CHC*H*_2_), 2.33-2.25 (m, 1H, *H*-2’), 2.10-1.99 (m, 1H, *H*-2’). ^13^C-NMR (125MHz, MeOD) δ 174.21, 174.01 (C=O ester), 163.58 (*C-*4), 152.02, 151.96, 151.88, 151.02 (*C-*2), 139.95, 139.72 (*C-*6), 138.13, 131.18, 130.88, 130.65, 130.55, 130.42, 129.66, 129.64, 128.97, 126.92, 126.35, 126.23, 121.61, 121.58, 121.49, 121.33, 121.29, 120.55, 116.29, 112.36, 112.32 (*C*-5, *Ar* Phe, *Ar* Ph, *C*-5a,), 109.51, 109.48 (*C*-5b), 87.15, 87.02, 86.82 (*C*-4’), 86.76, 86.71 *(C*-1’), 72.23, 72.13, 71.68 *(C*-3’), 67.56, 67.52, 67.49, 67.44 (*C*-5’), 62.56, 62.48, 61.61 (CH_3_*C*H_2_), 58.04, 57.95, 57.82 (CH_2_*C*H Phe), 41.43, 41.30, 41.20 *(C*-2’), 41.14, 41.07, 41.01 (*C*H_2_CH), 15.56, 14.55 (*C*H_3_CH_2_). MS [ESI, m/z]: 664.10 [M+H], 686.09 [M+Na]. t_R_ = 13.76 min.

**(E)-5-(2-bromovinyl)-2’-deoxyuridine-5’-(1-phenyl(cyclohexyloxy-L-valinyl)-phosphate (14)**

Purified by column chromatography (CH_2_Cl_2_/MeOH from 100/0 to 97/3), white solid, yield 21.6%.

^31^P NMR (202MHz, MeOD) δ 5.22, 4.76. ^1^H-NMR (500MHz; MeOD), δ 7.82-7.78 (m, 1H, *H*-6), 7.41-7.17 (m, 6H, Ar*H*, *H*-5b), 6.90-6.82 (m, 1H, *H*-5a), 6.31-6.22 (m, 1H, *H-*1’), 4.78-4.71 (m, 1H, *H*-1 cHex), 4.50-4.26 (m, 3H, *H*-5’, *H*-3’), 4.19-4.10 (m, 1H, *H*-4’), 3.73-3.61 (C*H*CO Val), 2.37-2.28 (m, 1H, *H*-2’), 2.18-2.01 (m, 2H, C*H*(CH_3_)_2_, *H*-2’), 1.91-1.69 (m, 4H, cHex), 1.61-1.52 (m, 1H, cHex), 1.49-1.27 (m, 5H, cHex), 1.01-0.84 (m, 6H, CH(C*H*_3_)_2_). ^13^C-NMR (125 MHz; MeOD), δ 173.44, 173.01 (C=O ester), 163.62 (*C-*4), 151.06, 151.02 (*C-*2), 146.99 (ipso Ph), 139.82, 139.77 (*C-*6), 130.84, 130.81, 130.54, 126.35, 126.27, 121.66, 121.62, 121.51, 121.47, 112.32 (*C*-2 Ph, *C*-3 Ph, *C*-4 Ph, *C*-5 Ph, *C*-6 Ph, *C*-5a, *C*-5), 109.32 (*C*-5b), 87.11, 87.01 (*C*-4’), 86.94, 86.81, 86.75 *(C*-1’), 75.00, 74.91 (*C*H-1 cHex), 72.29, 72.11 *(C*-3’), 67.95, 67.84, 67.79 (*C*-5’), 62.10, 62.03 (NH*C*H Val), 41.30, 41.27 *(C*-2’), 33.31, 33.06 ((*C*H_3_)_2_*C*H Val), 32.57, 26.40, 24.63 (*C*H_2_ cHex), 19.56, 18.45 ( (*C*H_3_)_2_). MS [ESI, m/z]: 670.14 [M+H], 692.12 [M+Na]. t_R_ = 16.09 min.

**(E)-5-(2-bromovinyl)-2’-deoxyuridine-5’-(1-phenyl(ethoxy-L-tyrosinyl) phosphate (15)**

Purified by column chromatography (CH_2_Cl_2_/MeOH from 100/0 to 97/3), white solid, yield 15.2 %. ^31^P NMR (202 MHz, MeOD) δ 4.06, 3.93. ^1^H-NMR (500 MHz, MeOD) δ 7.65 (s, 1H, *H*-6), 7.46-7.03 (m, 10H, *H*-5b, Ar*H),* 6.69-6.62 (m, 1H*, H*-5a), 6.31-6.24 (m, 1H, *H-*1’), 4.62-4.58 (m, 1H, *H*-5’), 4.53-4.49 (m, 1H, *H*-5’), 4.47-4.39 (m, 1H, *H*-3’), 4.18-4.09 (m, 4H, C*H*CH_2_, *H*-4’, C*H_2_*CH_3_), 3.11-3.02 (m, 1H, CHC*H*_2_), 2.92-2.84 (m, 1H, CHC*H*_2_), 2.37-2.30 (m, 1H, *H*-2’), 2.20-1.14 (m, 1H, *H*-2’), 1.28-1.24 (m, 3H, C*H*_3_). ^13^C-NMR (125 MHz, MeOD), δ 173.83 (C=O ester), 164.67 (*C-*4), 158.34, 158.26 (*C*-OH, Tyr), 150.76 (*C-*2), 147.89 (*ipso* Ph), 139.71, 139.67 (*C-*6), 136.35, 132.29, 132.21, 131.20, 131.14, 130.70, 130.37, 127.09, 125.98, 121.18, 116.03, 112.37 (*C*-1 Tyr*, C*-2 Tyr, *C*-3 Tyr, *C*-4 Tyr, *C*-5 Tyr, *C*-6 Tyr, *C*-2 Ph, *C*-3 Ph, *C*-5 Ph, *C*-6 Ph, *C*-5a, *C*-5), 109.26 (*C*-5b), 87.10, 86.84 *(C*-1’, *C*-4’), 71.62 *(C*-3’), 67.80, 67.68 (*C*-5’), 62.71, 62.58 (*C*H_2_ ester), 58.54, 58.41 (CH_2_*C*H Tyr), 40.15, 40.10 *(C*-2’), 38.28, 38.12 (*C*H_2_CH Tyr), 14.47 (*C*H_3_). MS [ESI, m/z]: 701.09 [M+Na]. t_R_ = 13.19 min.

**(E)-5-(2-bromovinyl)-2’-deoxyuridine-5’-(1-phenyl(cyclohexyloxy-L-alaninyl)-phosphate (16)**

Purified by column chromatography (CH_2_Cl_2_/MeOH from 100/0 to 97/3), white solid, yield 20.7%.

^31^P NMR (202MHz, MeOD) δ 4.30, 3.95. ^1^H-NMR (500 MHz; MeOD) δ 7.81 (s, 1H, *H*-6), 7.41-7.34 (m, 3H, Ar*H, H*-5b), 7.28-7.24 (m, 2H, Ar*H*), 7.23-7.19 (m, 1H, Ar*H*,), 6.87-6.82 (d, d, *J* = 13.8 Hz 1H, *H*-5a), 6.28-6.22 (m, 1H, *H-*1’), 4.78-4.71 (m, 1H, *H*-1 cHex), 4.42-4.29 (m, 3H, *H*-5’, *H*-3’), 4.17-4.10 (m, 1H, *H*-4’), 3.97-3.91 (C*H*CO Ala), 2.36-2.29 (m, 1H, *H*-2’), 2.12-2.07 (m, 1H, *H*-2’), 1.87-1.71 (m, 4H, cHex), 1.60-1.50 (m, 1H, cHex), 1.48-1.25 (m, 5H, cHex), 1.24-1.21 (m, 3H, C*H*_3_). ^13^C-NMR (125 MHz; MeOD), δ 173.44, 173.01 (C=O ester), 163.62 (*C-*4), 151.06, 151.02 (*C-*2), 146.99 (*ipso* Ph), 139.78 (*C-*6), 130.88, 130.52, 126.34, 21.55, 121.51, 112.32 (*C*-2 Ph, *C*-3 Ph, *C*-4 Ph, *C*-5 Ph, *C*-6 Ph, *C*-5a, *C*-5), 109.29 (*C*-5b), 87.14 (*C*-4’) 86.86, 86.79 *(C*-1’), 75.03 (*C*H-1 cHex), 72.14 *(C*-3’), 67.77, 67.73 (*C*-5’), 51.76 (*C*H Ala), 41.31 *(C*-2’), 32.49, 32.40, 26.40, 24.61 (*C*H_2_ cHex), 20.87, 20.68, 20.63 (*C*H_3_). MS [ESI, m/z]: 664.10 [M+Na]. t_R_ = 14.46 min.

**(E)-5-(2-carboxymethoxyvinyl)-2’-deoxyuridine-5’-(1-phenyl(methoxy-L-alaninyl)-phosphate (17)**

Purified by column chromatography (CH_2_Cl_2_/MeOH from 100/0 to 97/3), white solid, yield 7.6%.

^31^P NMR (202MHz, MeOD) δ 4.28, 3.86. ^1^H-NMR (500 MHz, MeOD) δ 8.01 (m, 1H, *H*-6), 7.4 (d, *J* = 13.8 Hz, 1H, *H*-5b), 7.39-7.34 (m, 2H, Ar*H*), 7.29-7.19 (m, 3H, Ar*H),* 6.96 (d, *J* = 13.8 Hz 1H, *H*-5a), 6.30-6.22 (m, 1H, *H-*1’), 4.46-4.35 (m, 1H, *H*-3’), 4.21-4.18 (m, 3H, *H*-5’, *H*-4’), 3.71 (s, 3H, C*H*_3_CO), 3.53-3.49 (m, 1H, C*H*CH_2_), 2.42-2.34 (m, 1H, *H*-2’), 2.20-1.13 (m, 1H, *H*-2’), 1.22-1.18 (m, 3H, C*H*_3_). ^13^C-NMR (125MHz, MeOD), δ 173.61 (C=O ester), 169.83 (*C*OC=C), 163.23 (*C-*4), 152.71 (*C-*2), 147.56 (*ipso* Ph), 144.46 (*C-*6), 142.09, 139.01, 137.31, 130.80, 126.27, 121.46, 118.98, (*C*-5, *C*-2 Ph, *C*-3 Ph, *C*-4 Ph, *C*-5 Ph, *C*-6 Ph, *C*-5a, *C*-5b), 87.68 *(C*-1’), 87.09 (*C*-4’), 72.17 *(C*-3’), 66.91 (*C*-5’), 54.80 (*C*H_3_OCO), 52.81 (*C*H_3_CO), 52.00 (CH_3_*C*H Ala), 41.50 *(C*-2’), 21.42 (CH*C*H_3_). MS [ESI, m/z]: 554.15 [M+H], 576.18 [M+Na], t_R_ = 14.42 min.

**(E)-5-(2-carboxymethoxyvinyl)-2’-deoxyuridine-5’-(1-phenyl(ethoxy-L-phenylalanine)-phosphate (18)**

Purified by column chromatography (CH_2_Cl_2_/MeOH from 100/0 to 97/3), white solid, yield 17.6 %. ^31^P NMR (202MHz, MeOD) δ 3.96, 3.82. ^1^H-NMR (500MHz; MeOD) δ 8.00 (s, 1H, *H*-6)*,* 7.49- 7.41 (m, 1H, *H*-5b), 7.33-7.08 (m, 10H, Ar*H)*, 7.03-6.95 (m, 1H, *H*-5a), 6.25-6.20 (m, 1H, *H-*1’), 4.40-4.29 (m, 1H, *H*-3’), 4.21-3.95 (m, 6H, *H*-4’, C*H*CH_2_, C*H_2_*CH_3_, *H*-5’), 3.72 (s, 3H, C*H*_3_), 3.12-3.08 (m, 1H, CHC*H*_2_), 2.93-2.88 (m, 1H, CHC*H*_2_), 2.38-2.29 (m, 1H, *H*-2’), 2.10-2.00 (m, 1H, *H*-2’), 1.20-1.16 (m, 3H, C*H*_3_). ^13^C-NMR (125 MHz, MeOD) δ 174.21 (C=O ester), 169.66 (*C*OC=C), 163.51 (*C-*4), 150.91 (*C-*2), 144.36, 144.33 (*ipso* OPh), 139.05 (*C-*6), 138.33, 138.21, 130.77, 130.64, 130.61, 129.59, 127.95, 126.19, 126.11, 121.56, 121.52, 121.23, 121.20, 118.99 (*C*-5, *Ar* Phe, *C*-2 Ph, *C*-3 Ph, *C*-4 Ph, *C*-5 Ph, *C*-6 Ph, *C*-5a,), 110.60 (*C*-5b), 87.56, 87.46 (*C*-4’), 87.12, 87.06, 86.96, 86.89 *(C*-1’), 72.12, 72.09 *(C*-3’), 67.50, 67.45, 67.36, 67.32 (*C*-5’), 62.43, 62.38 (CH_3_*C*H_2_), 57.99, 57.83 (CH_2_*C*H Phe), 52.04, 52.01 (*C*OC=C), 41.51 *(C*-2’), 41.10, 40.93, 40.86 (*C*H_2_CH), 14.39 (*C*H_3_CH_2_). MS [ESI, m/z]: 644.19 [M+H], 666.18 [M+Na]. t_R_ = 12.97 min.

**(E)-5-(2-carboxymethoxyvinyl)-2’-deoxyuridine-5’-(1-phenyl(methoxy-L-phenylalanine)-phosphate (19)**

Purified by column chromatography (CH_2_Cl_2_/MeOH from 100/0 to 97/3), white solid, yield 16.9 %.

^31^P NMR (202 MHz, MeOD) δ 3.90, 3.75. ^1^H-NMR (500 MHz, MeOD) δ 7.99 (s, 1H, *H*-6)*,* 7.41- 7.37 (m, 1H, *H*-5b), 7.32-7.06 (m, 10H, Ar*H)*, 7.01-6.92 (m, 1H, *H*-5a), 6.22-6.18 (m, 1H, *H-*1’), 4.40-4.30 (m, 1H, *H*-3’), 4.21-3.92 (m, 4H, *H*-4’, C*H*CH_2_, *H*-5’), 3.72 (s, 3H, C*H*_3_), 3.61 (s, 3H, C*H*_3_), 3.12-3.06 (m, 1H, CHC*H*_2_), 2.91-2.82 (m, 1H, CHC*H*_2_), 2.38-2.29 (m, 1H, *H*-2’), 2.10-1.99 (m, 1H, *H*-2’), 1.28-1.21 (m, 3H, C*H*_3_). ^13^C-NMR (125 MHz; MeOD) δ 174.60, 174.58 (C=O ester), 169.65, 169.61 (*C*OC=C), 163.51 (*C-*4), 150.91 (*C-*2), 144.35, 144.33 (*ipso* OPh), 139.04 (*C-*6), 138.27, 138.18, 132.41, 130.78, 130.63, 130.61, 130.39, 129.78, 129.63, 128.22, 127.99, 126.22, 126.13, 121.59, 121.56, 121.31, 121.24, 121.20, 119.01 (*C*-5, *Ar* Phe, *C*-2 Ph, *C*-3 Ph, *C*-4 Ph, *C*-5 Ph, *C*-6 Ph, *C*-5a,), 110.62, 110.60 (*C*-5b), 87.64, 87.46 (*C*-4’), 87.09, 87.03, 86.97, 86.90 *(C*-1’), 72.16, 72.08 *(C*-3’), 67.50, 67.46, 67.36, 67.32 (*C*-5’), 57.91, 57.78 (CH_2_*C*H *Phe*), 52.79, 52.77 (CH_3_ *Phe* ester), 52.12, 52.10 (CH_3_), 41.57, 41.50 *(C*-2’), 41.00, 40.94, 40.88 (*C*H_2_CH). MS [ESI, m/z]: 652.17 [M+Na], t_R_ = 11.76 min.

**(E)-5-(2-carboxymethoxyvinyl)-2’-deoxyuridine-5’-(1-phenyl(cyclohexyloxy-L-valinyl)-phosphate (20)**

Purified by column chromatography (CH_2_Cl_2_/MeOH from 100/0 to 97/3), white solid, yield 20.5%.

^31^P NMR (202MHz, MeOD) δ 5.19, 4.72. ^1^H-NMR (500 MHz; MeOD) δ 8.12-8.09 (m, 1H, *H*-6), 7.50-7.43 (m, 1H, *H*-5b), 7.38-7.31 (m, 2H, Ar*H*), 7.30-7.24 (m, 2H, Ar*H*), 7.22-7.18 (m, 1H, Ar*H*), 7.01-6.96 (m, 1H, *H*-5a), 6.28-6.21 (m, 1H, *H-*1’), 4.76-4.70 (m, 1H, *H*-1 cHex), 4.49-4.32 (m, 3H, *H*-5’, *H*-3’), 4.20-4.11 (m, 1H, *H*-4’), 3.74 (s, 3H, C*H*_3_ ester), 3.73-3.68 (C*H*CO Val), 2.39-2.32 (m, 1H, *H*-2’), 2.19-2.08 (m, 2H, C*H*(CH_3_)_2_, *H*-2’), 1.87-1.70 (m, 4H, cHex), 1.60-1.51 (m, 1H, cHex), 1.49-1.29 (m, 5H, cHex), 0.98-0.86 (m, 6H, CH(C*H*_3_)_2_). ^13^C-NMR (125 MHz; MeOD) δ 173.56, 173.41 (C=O ester), 169.56 (*C*OC=C), 164.72 (*C-*4), 152.13, 150.82 (*C-*2), 148.76 (ipso Ph), 139.03 (*C-*6), 130.80, 130.77, 130.29, 126.15, 125.97, 121.68, 121.60, 121.56, 121.37, 119.04 (*C*-2 Ph, *C*-3 Ph, *C*-4 Ph, *C*-5 Ph, *C*-6 Ph, *C*-5a, *C*-5), 110.35 (*C*-5b), 87.51 (*C*-4’), 86.89, 86.78, 86.71 *(C*-1’), 74.97, 74.89 (*C*H-1 cHex), 72.18, 72.05 *(C*-3’), 67.90, 67.82, 67.77 (*C*-5’), 63.20, 62.19 (NH*C*H Val), 52.00 (*C*H_3_OCO), 41.48 *(C*-2’), 33.48, 33.28 ((*C*H_3_)_2_*C*H Val), 32.55, 26.39, 24.62 (*C*H_2_ cHex), 19.52 ( (*C*H_3_)_2_). MS [ESI, m/z]: 672.23 [M+Na], t_R_ = 14.42 min.

**(E)-5-(2-carboxymethoxyvinyl)-2’-deoxyuridine-5’-(1-phenyl(benzyloxy-L-alaninyl)phosphate (21)**

Purified by column chromatography (CH_2_Cl_2_/MeOH from 100/0 to 97/3), white solid, yield 18.9 %.

^31^P NMR (202MHz, MeOD) δ 4.34, 3.78. ^1^H-NMR (500 MHz, MeOD) δ 8.08-8.03 (m, 1H, *H*-6), 7.39-7.30 (m, 6H, Ar*H*), 7.28-7.15 (m, 4H, Ar*H, H*-5b), 6.99-6.92 (m, 1H, Ar*H*), 6.82-6.76 (m, 1H, *H*-5a), 6.28-6.19 (m, 1H, *H*-1’), 5.13-5.10 (m, 2H, C*H*_2_Ph), 4.47-4.32 (m, 3H, *H*-3’, *H*-5’), 4.30-4.21 (m, 2H, CH_3_C*H*, *H*-4’), 3.71-3.68 (m, 3H, C*H_3_*), 2.38-2.30 (m, 1H, *H*-2’), 2.12-2.07(m, 1H, *H*-2’), 1.39-1.31 (m, 3H, C*H*_3_) ^13^C-NMR (125 MHz, MeOD) δ 173.63, 173.51 (C=O ester), 169.35 (*C*OC=C), 163.57 (*C-*4), 150.98 (*C-*2), 139.88 (*C-*6), 148.45, 136.26, 130.87, 130.62, 129.78, 129.35, 126.39, 121.67, 116.57 (*C*-5a, *C*-5*,* Ph), 109.79 (*C*-5b), 87.57, 86.86 *(C*-1’), 86.47 (*C*-4’), 72.63 *(C*-3’), 71.87 (Ph*C*H_2_ ester), 67.75 (*C*-5’), 51.96 (*C*H_3_OCO), 51.91 (CH_3_*C*H), 41.21 *(C*-2’), 20.52, 20.46 (*C*H_3_). MS [ESI, m/z]: 630.18 [M+H], 652.17 [M+Na], t_R_ = 13.48 min.

**(E)-5-(2-bromovinyl)-2’-deoxyuridine-5’-[1-naphthyl-(neopentyloxy-L-alaninyl)]phosphate (22)**

Purified by column chromatography (CH_2_Cl_2_/MeOH from 100/0 to 97/3), white solid, yield 3%.

^31^P-NMR (202MHz, CDCl_3_) δ 3.88, 3.62. ^1^H-NMR (500MHz, CDCl_3_) δ 8.51 (bs, 1H, H-3), 8.09 (d, *J* = 8.2 Hz, 1H, H-8 Nap), 7.81 (d, J = 8.2 Hz, 1H, H-5 Nap), 7.62 (m, 1H, H-6), 7.57-7.39 (m, 3H, H-2 Nap, H-3 Nap, H-4 Nap), 7.41-7.32 (m, 3H, H-6 Nap, H-7 Nap, H-5b), 6.69- 6.61 (d, J = 13.6 Hz, 1H, H-5a), 6.19-6.11 (m, 1H, H-1’), 4.59-4.31 (m, 4H, H- 3’, H-4’, H-5’), 4.21-4.11 (m, 1H, CH_3_C*H*), 4.00 (N*H*CH), 3.89 (m, 2H, C*H*_2_C(CH_3_)_3_), 3.77-3.53 (m, 2H, C*H*_2_C(CH_3_)_3_), 2.31-1.87 ( m, 1H, one of H-2a’), 1.47-1.37 (m, 3H, C*H*_3_CH), 0.97, 0.91 (s, 9H, CH_2_C(CH_3_)_3_), ^13^C-NMR (125MHz, CDCl_3_) δ 172.90 (C=O), 161.02, 160.92 (C-2), 148.86 (C- 4), 145.1 (*ipso* Nap), 139.56 (C-6), 137.24, 137.77 (C-4a Nap) 122.61, 126.20, 126.27, 126.48, 126.53, 127.55, 127.88, 128.92, 128.99, 130.46 (C-5a, C-3 Nap, C-4 Nap, C-5 Nap, C-6 Nap, C-7 Nap, C-8 Nap, C-8a Nap), 116.34, 116.60 (C-2 Nap), 114.49, 111.43 (C-5), 110.03 (C-5b), 86.91, 86.96, 87.19 (C-4’, C-1’), 74.88 (O*C*H_2_C), 72.21, 72.31 (C- 3’), 67.97, 68.05 (C-5’), 51.86, 51.94 (CHCH_3_), 41.16, 41.28 (C-2’), 26.30 (CH_2_C(*C*H_3_)_3_), 20.50, 20.71 (CH*C*H_3_); MS [ESI, m/z]: 702.56 [M+Na], t_R_ = 16.83 min.

**(E)-5-(2-bromovinyl)-2’-deoxyuridine-5’-[1-Naphthyl-(benzyloxy-L-alaninyl)]-phosphate (23)**

Purified by column chromatography (CH_2_Cl_2_/MeOH from 100/0 to 97/3), white solid, yield 17%. ^31^P-NMR (202MHz, CDCl_3_) δ 3.70, 3.41. ^1^H-NMR (500MHz, CDCl_3_) δ 8.13 (m, 1H, *H*-8 Nap), 7.64 (m, 2H, *H*-5 Nap, *H*-4 Nap), 7.56-7.43 (m, 4H, *H*-2 Nap, *H*-3 Nap, *H*-6 Nap, *H*-7 Nap), 7.39-7.23 (m, 7H, *H*-5b, *H*-6 and Ar*H*), 6.71-6.69 (d, *J* = 13.5 Hz, 1H, *H*-5a), 6.22-6.13 (m, 1H, *H*-1’), 5.12, 5.00 (2 s, 2H, C*H*_2_Ph), 4.49-4.37 (m, 4H, *H*-3’, *H*-4’, *H*-5’), 4.29 (m, 1H, C*H*CH_3_), 2.31-2.28 (m, 1H, *H*-2’), 1.91-1.82 (m, 1H, *H*-2’), 1.39-1.31 (m, 3H, C*H*_3_). ^13^C-NMR (125MHz, CDCl_3_) δ 173.46, 173.47 (C=O), 160.97, 161.01 (*C*-2), 148.85 (*C*-4), 146.95 (*ipso* Nap), 137.41, 137.46 (*C*-6), 137.07 (*ipso* Ph), 134.76 (*C*-4a Nap), 121.09, 122.59, 126.27, 126.52, 126.68, 127.08, 127.61, 127.64, 127.91, 128.75, 129.03, 129.25, 129.41, 129.54, 130.36 (*C*-5a, *C*-3 Nap, *C*-4 Nap, *C*-5 Nap, *C*-6 Nap, *C*-7 Nap, *C*-8 Nap, *C*-8a Nap), 115.26, 115.40 (d, *J* = 3.6 Hz, *C*-2 Nap), 111.44 (*C*-5), 110.00, 110.08 (*C*-5b), 85.30, 85.51 (*C*-1’), 84.86, 84.98 (d, *J* = 6.6 Hz, *C*-4’), 70.33, 70.71 (*C*-3’), 67.47 (*C*H_2_Ph), 66.01 (*C*-5’), 50.56, 50.66 (*C*HCH_3_), 40.20, 40.27 (*C*-2’), 20.57, 20.62 (*C*H_3_),. MS [ESI, m/z]: 722.08 [M+Na], t_R_ = 15.52 min.

**(E)-5-(2-bromovinyl)-2’-deoxyuridine-5’-[1-naphthyl(cyclohexyloxy-L-alaninyl)]-phosphate (24)**

Purified by column chromatography (CH_2_Cl_2_/MeOH from 100/0 to 97/3), white solid, yield 7.9 %. ^31^P-NMR (202MHz, CDCl_3_) δ 3.96, 3.66. ^1^H-NMR (500MHz, CDCl_3_) δ 9.47 (bs, 1H, *H*-3), 8.09 (d, 1H, *J* = 7.4 Hz, *H*-8 Nap), 7.79-7.81 (d, 1H, *J* = 7.2 Hz, *H*-5 Nap), 7.69 (m, 1H, *H*-4 Nap), 7.53-7.46 (m, 4H, *H*-2 Nap, *H*-3 Nap, *H*-6 Nap, *H*-7 Nap), 7.37-7.30 (m, 2H, *H*-5b, *H*-6), 6.69, 6.61 (d, 1H, *J* = 13.6 Hz, *H*-5a), 6.11 (m, 1H, *H*-1’), 4.75 (m, 1H, O*C*HCH_2_CH_2_), 4.62-4.29 (m, 3H, *H*-4’, *H*-5’), 4.20-4.07 (m, 2H, *H*-3’, C*H*CH_3_), 2.33 (m, 1H, *H*-2’), 1.99-1.60 (m, 7H, *H*-2’, 3xC*H*_2_), 1.50-1.19 (m, 7H, C*H*_3_CH, 2xC*H*_2_). ^13^C-NMR (125MHz, CDCl_3_) δ 172.90 (*C*=O), 160.35, 161.38 (*C*-2), 149.19 (*C*-4), 145.1 (*ipso* Nap), 137.43 (*C*-6), 134.75 (*C*-4a Nap), 121.18, 125.24, 125.44, 125.55, 126.29, 126.53, 126.66, 126.75, 126.83, 127.97, 128.31 (*C*-5a, *C*-3 Nap, *C*-4 Nap, *C*-5 Nap, *C*-6 Nap, *C*-7 Nap, *C*-8 Nap, *C*-8a Nap), 115.20, 115.38 (*C*-2 Nap), 111.48 (*C*-5b), 110.33 (*C*-5), 85.22, 85.40, 85.56 (*C-*4’, *C*-1’), 74.37 (O*C*HCH_2_CH_2_), 70.59, 70.87 (*C*-3’), 66.16 (*C*-5’), 50.64 (*C*HCH_3_), 40.16, 40.29 (*C*-2’), 31.27, 31.37 (*C*H_2_), 25.19 (*C*H_2_), 23.47 (*C*H_2_), 20.96, 21.00 (*C*H_3_), MS [ESI, m/z] 714.11 [M+Na]. t_R_ = 17.12min.

**(E)-5-(2-bromovinyl)-2’-deoxyuridine-5’-[1-naphthyl(ethyloxy-L-alaninyl)]phosphate (25)**

Purified by column chromatography (CH_2_Cl_2_/MeOH from 100/0 to 97/3), white solid, yield 14 %.

^31^P-NMR (202MHz, CDCl_3_) δ 4.97, 4.89. ^1^H-NMR (500MHz, CDCl_3_) δ 8.03-8.00 (1H, m, H-8 Nap), 7.79-7.76 (m, 1H, H-5 Nap), 7.61-7.59 (m, 1H, H-4 Nap), 7.52-7.42 (m, 4H, H-2 Nap, H-3 Nap, H-6 Nap, H-7 Nap), 7.35-7.28 (m, 2H, H-5b, H-6), 6.61-6.54 (d, *J* = 13.6 Hz, 1H, H-5a), 6.23-6.18 (m, 1H, H-1’), 4.99 (m, 1H, C*H*CH_3_), 4.43-4.26 (m, 4H, H-3’, H-4’, H-5’), 4.18-3.95 (m, 4H, OC*H*_2_CH_3_, OH- 3’, CHN*H*), 2.38-1.86 (m, 1H, H-2’), 1.43-1.20 (m, 6H, CHC*H*_3_, OCH_2_C*H*_3_); ^13^C-NMR (125MHz, CDCl_3_) δ 172.90 (C=O), 161.02, 160.92 (C-2), 148.3 (C-4), 145.1 (ipso-Nap), 137.43 (C-6), 137.24, 137.77 (C-4a Nap), 115.24, 115.44 (C-2 Nap), 128.32, 128.25, 128.00, 126.85, 126.76, 126.67, 126.53, 126.30, 125.54, 124.45, 125.28, 121.14, 121.09 (C-5a, C-3 Nap, C-4 Nap, C-5 Nap, C-6 Nap, C-7 Nap, C-8 Nap, C-8a Nap), 114.44, 115,49 (C-5), 109.0 (C-5b), 84.4, 84.6 (C-1’), 84.1, 84.2 (C-4’), 69.60, 69.60 (C-3’), 65.20 (C-5’), 60.80, 60.90 (OCH_2_CH_3_), 49.40, 49.50 (*C*HCH_3_), 39.10, 39.30 (C-2’), 19.80, 19.90 (*C*HCH_3_), 13.00 (OCH_2_*C*H_3_). MS [ESI, m/z]: 661.21 [M+Na]. t_R_ = 13.95 min.

**(E)-5-(2-bromovinyl)-2’-deoxyuridine-5’-[1-naphthyl(isopropyloxy-L-alaninyl)]phosphate (26)**

Purified by column chromatography (CH_2_Cl_2_/MeOH from 100/0 to 97/3), white solid (yield 28 %). ^31^P-NMR (202MHz, CDCl_3_) δ 3.84, 3.64, ^1^H-NMR (500MHz, CDCl_3_) δ 10.01 (bs, 1H, *H*-3), 8.19 (m, 1H, *H*-8 Nap), 7.79-7.91 (m, 1H, *H*-5 Nap), 7.69-7.67 (m, 1H, *H-*4 Nap), 7.59-7.49 (m, 4H, *H*-2 Nap, *H*-3 Nap, *H*-6 Nap, *H*-7 Nap), 7.39-7.25 (m, 2H, *H*-5b, *H*-6), 6.71-6.58 (d, *J* = 13.6 Hz, 1H, *H*-5a), 6.29-6.18 (m, 1H, *H*-1’), 4.99 (m, 1H, (CH_3_)_2_C*H*), 4.62-4.39 (m, 4H, *H*-3’, *H*-4’, *H*-5’), 4.29 (m, 1H, C*H*CH_3_), 4.19-3.99 (m, 1H, NH), 2.48-2.45 (m, 1H, *H*-2’), 1.89-1.72 (m, 1H, *H*-2’), 1.39 (m, 3H, C*H*_3_CH), 1.29-1.08 (m, 6H, (C*H*_3_)_2_CH), ^13^C-NMR (125MHz, CDCl_3_) δ 172.90 (*C*=O), 161.02, 160.92 (*C*-2), 148.86 (*C*-4), 145.1 (*ipso* Nap), 137.43 (*C*-6), 137.24, 137.77 (*C*-4a Nap), 128.32, 128.25, 128.00, 126.85, 126.76, 126.67, 126.53, 126.30, 125.54, 124.45, 125.28, 121.14, 121.09 (*C*-5a, *C*-3 Nap, *C*-4 Nap, *C*-5 Nap, *C*-6 Nap, *C-*7 Nap, *C*-8 Nap, *C*-8a Nap), 115.44, 115.24 (*C*-2 Nap), 114.49, 111.43 (*C*-5), 110.03 (*C*-5b), 84.4, 84.6 (*C*-1’), 84.1, 84.2 (*C*-4’), 70.43, 70.84 (*C*-3’), 69.66 (*C*H(CH_3_)_2_), 65.97 (*C*-5’), 50.60, 50.65 (*C*HCH_3_), 40.20, 40.31 (*C*-2’), 21.60, 21.67 (CH(*C*H_3_)_2_), 20.82, 20.86 (CH*C*H_3_). MS [ESI, m/z]: 674.09 [M+Na]. t_R_ = 13.82min.

**(E)-5-(2-bromovinyl)-2’-deoxyuridine-5’-[1-naphthyl(benzyloxy-L-methionyl)]phosphate (27)**

Purified by column chromatography (CH_2_Cl_2_/MeOH from 100/0 to 97/3), white solid, yield 5 %. ^31^P-NMR (202MHz, CDCl_3_) δ 5.34, 5.22. ^1^H-NMR (500MHz, CDCl_3_) δ 8.21-8.13 (m, 1H, *H*-8 Nap), 7.87-7.84 (m, 1H, *H*-5 Nap), 7.70-7.64 (m, 2H, *H*-6, *H*-4 Nap), 7.57-7.21 (m, 9H, *H*-2 Nap, *H*-3 Nap, *H*-6 Nap, *H*-7 Nap, *H*-5b, Ar*H*), 6.81-6.68 (m, 1H, *H*-5a), 6.18, 6.11 (m, 1H, *H*-1’), 5.12-5.00 (m, 2H, C*H*_2_Ph), 4.51-4.31 (m, 3H, *H*-4’, *H*-5’), 4.21-4.08 (m, 2H, *H*-3’, C*H*CH_2_), 3.85 (N*H*CH), 2.49-2.36 (m, 2H, C*H*_2_S), 2.28-2.15 (m, 1H, *H-*2’), 2.00-1.91 (m, 5H, C*H*_2_CH_2_S, SC*H*_3_), 1.90-1.83 (m, 1H, *H*-2’); ^13^C-NMR (125MHz, CDCl_3_) δ 173.61 (*C*=O), 162.37 (*C*-2), 150.13 (*C*-4), 147.12 (*ipso* Nap), 140.01, 140.11 (*C*-6), 137.45, 137.58 (*C*-4a Nap), 134.73, 135.09 (*ipso* Ph), 112.28, 112.36, 123.08, 123.15, 126.23, 126.53, 127.28, 127.72, 127.88, 128.81, 129.11, 129.32, 129.47, 129.67, 129.93 (*C*-5, , *C*-5a, *C*-2 Nap, *C*-3 Nap, *C*-4 Nap, *C*-5 Nap, *C*-6 Nap, *C*-7 Nap, *C-*8 Nap, *C*-8a Nap, *C*-2 Ph, *C*-3 Ph, *C*-4 Ph, *C*-5 Ph, *C*-6 Ph), 109.44 (*C*-5b), 86.94, 86.99, 87.29, 87.48 (*C*-4’, *C*-1’), 72.39, 72.65 (*C*-3’), 68.07, 68.10 (*C*-5’), 51.25, 54.18 (NHC*H*), 41.16, 41.23 (*C*-2’), 34.02, 34.13, 34.56, 34.61 (*C*H_2_CH_2_S), 31.32 (*C*H_2_S), 15.51, 15.53 (S*C*H_3_), MS [ESI, m/z]: 760.20 [M + H], t_R_ = 18.36 min.

**(E)-5-(2-bromovinyl)-2’-deoxyuridine-5’-[1-naphthyl(benzyloxy-L-valinyl)]phosphate (28)**

Purified by column chromatography (CH_2_Cl_2_/MeOH from 100/0 to 97/3), white solid, yield 10 %. ^31^P-NMR (202MHz, CDCl_3_) δ 4.89, 4.44. ^1^H-NMR (500MHz, CDCl_3_) δ 9.01 (bs, 1H, *H*-3), 8.11 (m, 1H, *H*-8 Nap), 7.81 (m, 1H, *H*-5 Nap), 7.73 (t, *J* = 8.2 Hz, 1H, *H*-4 Nap), 7.59-7.49 (m, 4H, *H*-2 Nap, *H*-3 Nap, *H*-6 Nap, *H*-7 Nap), 7.39-7.25 (m, 7H, *H*-5b, *H*-6 and Ar *H*), 6.67 (d, *J* = 13.6 Hz, 1H, *H*-5a), 6.19, 6.11 (m, 1H, *H*-1’), 5.11 (m, 2H, C*H*_2_Ph), 4.99 (s, 1H, CH_2_Ph), 4.48-4.27 (m, 3H, *H*-4’, *H*-5a’ and *H*-5b’), 4.09 (m, 1H, *H*-3’), 3.99, 3.92 (m, 1H, N*H*CH), 2.11 (m, 1H, *H*-2’), 1.79 (m, 2H, H-2’, C*H*(CH_3_)_2_), 0.81 (m, 6H, CH(C*H*_3_)_2_); ^13^C-NMR (125MHz, CDCl_3_) δ 172.91, 172.91 (*C*=O), 161.13 (*C*-2), 149.00 (*C*-4), 137.43 (*ipso* Ph), 134.73 (*C*-6), 134.71 (*C*-4a Nap), 121.01, 125.24, 125.32, 125.46, 126.54, 126.75, 126.83, 127.99, 128.25, 128.34, 128.44, 128.51, 128.57, 128.61, 128.67 (*C*-3 Nap, *C*-4 Nap, *C*-5 Nap, *C*-6 Nap, *C*-7 Nap, *C*-8 Nap, *C*-8a Nap, *C*-5a, *C*-2 Ph, *C*-3 Ph, *C*-4 Ph, *C*-5 Ph, *C*-6 Ph), 115.34 (*C*-2 Nap), 111.52 (*C-*5), 110.03 (*C*-5b), 85.27, 85.50 (*C*-1’), 84.88, 85.11 (*C*-4’), 70.50, 70.87 (*C*-3’), 67.26 (*C*H_2_Ph), 66.10 (*C*-5’), 60.36 (d, *J =* 6.7 Hz, NH*C*H), 40.15 (*C*-2’), 32.18, 32.20 (*C*H (CH_3_)_2_), 17.20, 17.28 (CH(*C*H_3_)_2_). MS [ESI, m/z]: 750.11 [M+Na], t_R_ = 17.12 min.

**(E)-5-(2-bromovinyl)-2’-deoxyuridine-5’-[1-naphthyl(benzyloxy-L-prolinyl)]phosphate (29)**

Purified by column chromatography (CH_2_Cl_2_/MeOH from 100/0 to 97/3), white solid, yield 18 %. ^31^P-NMR (202MHz, CDCl_3_) δ 2.70, 2.37. ^1^H-NMR (500MHz, CDCl_3_) δ 9.31 (bs, 1H, *H*-3), 8.11 (d, *J* = 8.4 Hz, 1H, *H*-8 Nap), 7.83 (d, *J* = 8.6 Hz, 1H, *H-*5 Nap), 7.67 (d, *J* = 8.2 Hz, 1H, *H*-4 Nap), 7.65-7.51 (m, 4H*, H*-2 Nap, *H*-3 Nap, *H*-6 Nap, *H*-7 Nap), 7.43-7.29 (m, 7H, *H*-5b, *H*-6 and Ar *H*), 6.71 (d, *J* = 13.6 Hz, 1H, *H*-5a), 6.25, 6.13 (m, 1H, *H*-1’), 5.27, 5.00 (d, *J* = 12.3 Hz, 2H, CH_2_Ph), 4.69-4.39 (m, 4H, *H*-3’, *H*-4’, *H*-5’), 4.12 (m, 1H, NC*H*CO_2_), 3.71-3.40 (m, 1H, C*H*_2_NH), 3.57-3.30 (m, 1H, C*H*_2_NH), 2.31 (m, 1H, C*H*_2_-CH-CO_2_), 1.98-1.89 (5H, m, C*H*_2_-CH-CO_2_, CH_2_C*H*_2_CH_2_NH, *H*-2’). ^13^C-NMR (125MHz, CDCl_3_) δ 173.65 (*C*=O), 161.32 (*C*-2), 149.13 (*C*-4), 146.29 (*ipso* Nap), 137.49 (*C*-6), 135.39 (*ipso* Ph), 134.77 (*C*-4a Nap), 120.94, 125.07, 125.46, 126.18, 126.48, 126.87, 127.98, 128.12, 128.20, 128.44, 128.50, 128.64, (*C*-5a, *C*-3 Nap, *C*-4 Nap, *C*-5 Nap, *C*-6 Nap, *C*-7 Nap, *C*-8 Nap, *C*-8a Nap, *C*-2 Ph, *C*-3 Ph, *C*-4 Ph, *C*-5 Ph, *C*-6 Ph), 114.52 (*C*-2 Nap), 111.37 (*C*-5), 109.90 (*C*-5b), 84.48, 85.53 (*C*-1’), 85.27 (*C*-4’), 70.55 (*C*-3’), 67.07, 67.23 (*C*H_2_Ph), 66.00 (*C*-5’), 61.25 (d, ^2^*J*_C-P_ = 5.5 Hz, -C*H*NH), 46.97 (d, ^3^*J*_C-P_ = 4.8 Hz, -*C*H_2_-NH), 40.26 (*C*-2’), 30.95 (d, ^3^*J*_C-P_ = 8.7 Hz, -C*H*_2_CHCO_2_CH_2_Ph), 25.19 (d, ^3^*J*_C-P_ = 8.7 Hz, -*C*H_2_-CH_2_-NH); MS [ESI, m/z]: 748.09 [M+Na], t_R_ = 17.09 min.

**(E)-5-(2-bromovinyl)-2’-deoxyuridine-5’-[1-naphthyl(2-butyloxy-L-alaninyl)]phosphate (30)**

Purified by column chromatography (CH_2_Cl_2_/MeOH from 100/0 to 97/3), white solid, yield 12 %. ^31^P-NMR (202MHz, CDCl_3_) δ 3.76, 3.64. ^1^H-NMR (500MHz, CDCl_3_) δ 9.70 (bs, 1H, *H*-3), 8.10 (d, *J* = 8.7 Hz, 1H, *H*-8 Nap), 7.80 (d, *J* = 8.7 Hz, 1H, *H-*5 Nap), 7.64 (m, 1H, *H*-4 Nap), 7.57-7.47 (m, 4H, *H*-2 Nap, *H*-3 Nap, *H*-6 Nap, *H*-7 Nap), 7.37-7.31 (m, 2H, *H*-5b, *H*-6), 6.68, 6.59 (d, *J* = 13.6 Hz, 1H, *H*-5a), 6.19 (m, 1H, *H*-1’), 4.81 (m, 1H, C*H*CH_3_), 4.51-4.29 (m, 4H, *H*-3’, *H*-4’, *H*-5’), 4.20 (m, 1H, CH_3_C*H*), 4.01 (m, 1H, N*H*), 2.37, 1.82 (*H*-2’), 1.52 (m, 2H, C*H*_2_CH_3_), 1.32 (m, 3H, C*H*_3_CH), 1.19 (m, 3H, CHC*H*_3_), 0.81 (m, 3H, CH_2_*C*H_3_); ^13^C-NMR (125MHz, CDCl_3_) δ 173.29 (*C*=O), 161.44, 161.52 (*C*-2), 149.29 (*C*-4), 146.25, 146.30 (*ipso*-Nap), 9.52, 9.55 (CH_2_*C*H_3_), 19.26 (d, *J* = 6.3 Hz, CH*C*H_3_), 20.93 (d, *J* = 5.7 Hz, CH*C*H_3_), 28.54, 28.60 (*C*H_2_CH_3_), 40.14, 40.30 (*C*-2’), 50.59, 50.64 (d, *J* = 5.7 Hz, *C*HCH_3_), 66.26 (*C*-5’), 70.66, 70.93 (*C*-3’), 74.13, 74.15 (d, *J* = 5.6 Hz, *C*HCH_3_), 85.06, 85.11, 85.23 (*C*-4’, *C*-1’), 110.03 (*C*-5), 111.55 (*C*-5b), 115.18, 115.37 (*C*-2 Nap), 121.15, 125.24, 125.45, 126.29, 126.51, 126.62, 126.73, 126.81, 127.94, 128.37, 128.36, (*C*-5a, *C-*3 Nap, *C*-4 Nap, *C*-5 Nap, *C*-6 Nap, *C*-7 Nap, *C*-8 Nap, *C*-8a Nap), 134.74 (*C*-4a Nap), 137.32, 137.54 (*C*-6), 137.32, 137.54 (*C*-6), 134.74 (*C*-4a Nap), 121.15, 125.24, 125.45, 126.29, 126.51, 126.62, 126.73, 126.81, 127.94, 128.37, 128.36, (*C*-5a, *C-*3 Nap, *C*-4 Nap, *C*-5 Nap, *C*-6 Nap, *C*-7 Nap, *C*-8 Nap, *C*-8a Nap), 115.18, 115.37 (*C*-2 Nap), 111.55 (*C*-5b), 110.03 (*C*-5), 85.06, 85.11, 85.23 (*C*-4’, *C*-1’), 74.13, 74.15 (d, *J* = 5.6 Hz, *C*HCH_3_), 70.66, 70.93 (*C*-3’), 66.26 (*C*-5’), 50.59, 50.64 (d, *J* = 5.7 Hz, *C*HCH_3_), 40.14, 40.30 (*C*-2’), 28.54, 28.60 (*C*H_2_CH_3_), 20.93 (d, *J* = 5.7 Hz, CH*C*H_3_), 19.26 (d, *J* = 6.3 Hz, CH*C*H_3_), 9.52, 9.55 (CH_2_*C*H_3_); MS [ESI, m/z]: 688.11 [M+Na]. t_R_ = 16.39min

**(E)-5-(2-bromovinyl)-2’-deoxyuridine-5’-[1-naphthyl(benzyloxy-glycinyl)]phosphate (31)**

Purified by column chromatography (CH_2_Cl_2_/MeOH from 100/0 to 97/3), white solid, yield 7.8 %. ^31^P-NMR (202MHz, CDCl_3_) δ 4.72, 4.44. ^1^H-NMR (500MHz, CDCl_3_): δ 8.09 (m, 1H, *H*-8 Nap), 7.85 (m, 1H, *H*-5 Nap), 7.69 (m, 1H, *H*-4 Nap), 7.56-7.49 (m, 4H, *H*-2 Nap, *H*-3 Nap, *H*-6 Nap, *H*-7 Nap), 7.41-7.27 (m, 7H, *H*-5b, *H*-6, *C*-2 Ph, *C*-3 Ph, *C*-4 Ph, *C*-5 Ph, *C*-6 Ph), 6.71, 6.72 (d, *J* = 13.5 Hz, 1H, *H*-5), 6.20 (m, 1H, *H*-1’), 5.19-5.11 (s, 1H, C*H*_2_Ph), 4.57-4.39 (m, 3H, H-4’, H-5’), 4.11 (m, 1H, *H*-3’), 4.05-3.90 (m, 1H, NHC*H*_2_), 3.89-3.77 (m, 1H, NHC*H*_2_), 3.75 (m, 1H, N*H*CH_2_), 2.41-2.30 (m, 1H, *H*-2’), 2.00, 1.91 (m, 1H, *H*-2’); MS [ESI, m/z]: 709.86 [M+Na]. t_R_ = 15.28 min.

**(E)-5-(2-bromovinyl)-2’-deoxyuridine-5’-[1-naphtyl(benzyloxy-D-alaninyl)]phosphate (32)**

Purified by column chromatography (CH_2_Cl_2_/MeOH from 100/0 to 97/3), white solid, yield 3.6 %.

^31^P-NMR (CDCl_3_, 202MHz): δ 2.81, 2.62. ^1^H-NMR (CDCl_3_, 500MHz): δ 8.13 (m, 1H, *H*-8 Nap), 7.99-7.91 (m, 1H, *H*-5 Nap), 7.69 (m, 1H, *H*-4 Nap), 7.59-7.49 (m, 4H, *H*-2 Nap, *H*-3 Nap, *H*-6 Nap, *H-*7 Nap), 7.39-7.25 (m, 7H, *H*-5b, *H*-6 and *C*-2 Ph, *C*-3 Ph, *C*-4 Ph, *C*-5 Ph, *C*-6 Ph), 6.71 (d, *J* = 13.6 Hz, 1H, *H*-5), 6.29 (m, 1H, *H*-1’), 5.29-5.03 (m, 2H, C*H*_2_Ph), 4.25-3.71 (m, 5H, *H*-3’, *H*-4’, *H*-5’, C*H*CH_3_), 3.75 (m, 1H, N*H*), 2.51-2.40 (m, 1H, *H*-2’), 1.80-1.73 (m, 1H, *H*-2’), 1.31 (m, 3H, C*H*_3_CH); ^13^C-NMR (125MHz, CDCl_3_) δ 172.90 (C=O), 161.02, 160.92 (*C*-2), 148.86 (*C*-4), 145.1 (*ipso* Nap), 138.20 (*C*-6), 137.50 (*ipso* Ph), 137.24, 137.77 (*C*-4a Nap), 121.33, 121.41, 125.20, 125.52, 126.60, 126.79, 127.92, 128.13, 128.34, 128.50, 128.57, 128.62 (*C*-5a, *C*-3 Nap, *C*-4 Nap, *C*-5 Nap, *C*-6 Nap, *C*-7 Nap, *C*-8 Nap, *C*-8a Nap, *C*-2 Ph, *C*-3 Ph, *C*-4 Ph, *C*-5 Ph, *C*-6 Ph), 115.33 (*C*-2 Nap), 114.49, 111.43 (*C*-5), 110.03 (*C*-5b), 85.58, 85.71, 86.00 (*C-*4’, *C*-1’), 74.91, 75.03 (*C*-3’), 67.45 (*C*H_2_Ph), 61.25, 61.45 (*C*-5’), 50.43, 50.49 (C*H*CH_3_), 39.23 (*C*-2’), 20.86 (CH*C*H_3_). MS [ESI, m/z]: 723.99 [M+Na], t_R_ = 16.79min

**(E)-5-(2-bromovinyl)-2’-deoxyuridine-5’-[1-naphthyl(cyclohexyloxy-L-valinyl)] phosphate (33)**

Purified by column chromatography (CH_2_Cl_2_/MeOH from 100/0 to 97/3), white solid, yield 9.8 %. ^31^P-NMR (202MHz, CDCl_3_) δ 5.06, 4.75. ^1^H-NMR (500MHz, CDCl_3_) δ 8.07 (m, 1H, *H*-8 Nap), 7.81 (m, 1H, *H*-5 Nap), 7.63 (m, 1H, *H*-4 Nap), 7.59-7.47 (m, 4H, *H*-2 Nap, *H*-3 Nap, *H*-6 Nap, *H*-7 Nap), 7.40-7.33 (m, 2H, *H*-5b, *H*-6), 6.69, 6.52 (d, *J* = 13.6 Hz, 1H, *H*-5), 6.13 (m, 1H, H-1’), 4.82-4.68 (m, 1H, C*H*CH_2_CH_2_), 4.49-4.30 (m, 3H, *H*-4’, *H*-5’), 4.00-3.80 (m, 2H, *H*-3’, NHC*H*), 3.75 (m, 1H, N*H*CH), 2.31 (m, 1H, *H*-2’), 2.17-1.92 (m, 3H, C*H*_2_CH_2_, C*H*(CH_3_)_2_)), 1.90-1-70 (m, 3H, *H*-2’, CH_2_C*H*_2_), 1.48-1.21 (m, 2H, CH_2_CH_2_), 1.00-0.76 (m, 6H, (C*H*_3_)_2_CH); ^13^C-NMR (125MHz, CDCl_3_) δ 172.20 (C=O), 161.32, 161.38 (*C*-2), 149.13, 149.19 (*C*-4), 137.74, 137.50 (*C*-6), 134.74 (*C*-4a Nap), 121.13, 121.23, 124.58, 125.25, 125.56, 126.48, 126.72, 127.76, 127.94, 128.31, 128.39 (*C*-3 Nap, *C*-4 Nap, *C*-5 Nap, *C*-6 Nap, *C*-7 Nap, *C*-8 Nap*, C*-8a Nap, *C*-5a), 115.35 (*C*-2 Nap), 111.47 (*C*-5), 110.05 (*C*-5b), 85.05, 85.31, 85.52 (*C*-4’, *C*-1’), 70.41, 70.77 (*C*-3’), 66.19 (*C*-5’), 60.06, 60.13, 60.28, 60.40 (d, *J =* 6.7 Hz, NH*C*H), 40.11, 40.25 (d, *J* = 5.5 Hz, *C-*2’), 32.32, 32.37 ((CH_3_)_2_*C*H), 31.42, 31.46 (*C*H_2_CH_2_), 25.18 (*C*H_2_CH_2_), 23.54 (*C*H_2_CH_2_), 18.74, 18.84 ((*C*H_3_)_2_CH), 17.07, 17.25 ((*C*H_3_)_2_CH); MS [ESI, m/z]: 742.14 [M+Na]. t_R_ = 14.68 min.

**(E)-5-(2-bromovinyl)-2’-deoxyuridine-5’-[1-naphthyl(ethyloxy-L-valinyl)]phosphate (34)**

Purified by column chromatography (CH_2_Cl_2_/MeOH from 100/0 to 97/3), white solid, yield 9.4 %. ^31^P-NMR (202MHz, CDCl_3_) δ 4.99, 4.66. ^1^H-NMR (500 MHz, CDCl_3_) δ 8.09 (m, 1H, *H*-8 Nap), 7.83 (, d, *J* = 12.5 Hz, 1H, *H*-5 Nap), 7.69 (m, 1H, *H*-4 Nap), 7.60-7.52 (m, 4H, *H*-2 Nap, *H*-3 Nap, *H*-6 Nap, *H*-7 Nap), 7.45-7.35 (m, 2H, *H*-5b, *H*-6), 6.71, 6.73 (d, *J* = 13.6 Hz, 1H, *H*-5a), 6.19 (m, 1H, *H*-1’), 4.50-4.30 (m, 3H, *H*-4’, *H*-5’), 4.17-4.10 (m, 3H, *H*-3’, C*H*_2_CH_3_), 4.06-3.92 (m, 1H, NHC*H*), 3.88, 3.77 (m, 1H, N*H*CH), 2.32 (m, 1H, m, *H*-2’), 1.92-1.80 (m, 1H, *H*-2’), 1.22 (t, *J* = 10.5 Hz, 3H, C*H*_3_CH_2_), 1.20 (t, *J* = 10.5 Hz, 3H, C*H*_3_CH_2_), 0.89 (m, 3H, CH(CH_3_)), 0.80 (m, 3H, CH(CH_3_)), ^13^C-NMR (125MHz, CDCl_3_) δ 172.90 (*C*=O), 161.02, 160.92 (*C*-2), 148.86 (*C*-4), 145.32 (*ipso* Nap), 137.43, 137.52 (*C*-6), 134.75 (*C*-4a Nap), 121.04, 121.17, 125.21, 125.28, 125.46, 126.51, 126.69, 126.74, 126.83, 127.98, 128.26, 128.33 (*C*-5a, *C*-3 Nap, *C*-4 Nap, *C*-5 Nap, *C*-6 Nap, *C*-7 Nap, *C*-8 Nap, *C*-8a Nap), 115.33 (*C*-2 Nap), 111.53 (*C*-5), 110.03 (*C*-5b), 85.25, 85.51 (*C*-4’, *C*-1’), 70.41, 70.80 (*C*-3’), 66.13, 66.21 (d, *J* = 3.5 Hz, *C*-5’), 61.57 (*C*H_2_CH_3_), 60.18, 60.31 (d, *J =* 6.7 Hz, NH*C*H), 40.18, 40.27 (J = 3.2 Hz, *C*-2’), 31.84, 32.18 ((CH_3_)_2_*C*H), 18.83, 18.87 ((*C*H_3_)_2_CH), 17.08, 17,35 ((*C*H_3_)_2_CH), 14.07, 14.14 (d, *J* = 7.5 Hz, CH_2_*C*H_3_). MS [ESI, m/z]: 688.10 [M+Na]. t_R_ = 15.31min.

**(E)-5-(2-bromovinyl)-2’-deoxyuridine***-5’-***(1-naphthyl(methoxy-L-tryptophanyl)-phosphate (35f)**

Purified by column chromatography (CH_2_Cl_2_/MeOH from 100/0 to 97/3), white solid, yield 11.6%

^31^P NMR (202 MHz, MeOD) δ 4.55. ^1^H-NMR (500 MHz; MeOD) δ 8.08-8.06 (m, 1H, Ar*H*), 7.86-7.83 (m, 1H, Ar*H*), 7.65 (d, *J* = 8 Hz, 1H, Ar*H*), 7.55 (s, 1H, *H*-6), 7.52-7.47 (m, 3H, Ar*H),* 7.39-7.30 (m, 3H, Ar*H*), 7.25 (d, *J* = 13.5 Hz, 1H, *H*-5b), 7.08-7.05 (m, 2H, Ar*H*), 6.99-6.96 (m, 1H, Ar*H*), 6.63 (d, *J* = 13.5 Hz, 1H, *H*-5a), 6.09-6.06 (m, 1H, *H-*1’), 4.36-4.31 (m, 1H, C*H*CH_2_-Trp), 4.21-4.20 (m, 1H, *H*-3’), 4.04-3.99 (m, 1H, *H*-5’,), 3.95-3.93 (m, 1H, *H*-4’), 3.87-3.84 (m, 1H, *H*-5’), 3.57 (s, 3H, C*H*_3_), 3.26-3.22 (m, 1H, CHC*H_2_*-Trp), 3.10-3.06 (m, 1H, CHC*H_2_*-Trp), 2.17-2.14 (m, 1H, *H*-2’), 1.72-1.67 (m, 1H, *H*-2’). ^13^C-NMR (125 MHz; MeOD), δ 174.93 (d, ^3^*J*_C-P_ = 2.5 Hz, C=O ester), 163.54 (*C-*4), 150.88 (*C-*2), 147.67 (d, ^2^*J*_C-P_ = 6.25 Hz, *ipso* Nap), 139.37 (*C-*6), 138.13, 137.97, 136.25, 130.39, 128.93, 128.73, 127.85, 127.82, 127.54, 126.51, 126.21, 125.01, 124.84, 122.64, 122.53, 119.97, 119.22, 116.54, 112.44, 112.17, 110.73, 110.68, (*C*-5, *C*-2 Nap, *C*-3 Nap, *C*-4 Nap, *C*-4a Nap, *C*-5 Nap, *C*-6 Nap, *C*-7 Nap, *C*-8 Nap, *C*-8a Nap, *C*-2 Trp, *C*-3 Trp, *C*-3a Trp, *C*-4 Trp, *C*-5 Trp, *C*-6 Trp, *C*-7 Trp, *C*-7a Trp, *C*-5a), 109.21 (*C*-5b), 87.15 (*C*-4’), 86.77 *(C*-1’), 72.22 *(C*-3’), 67.58 (d, ^2^*J*_C-P_ = 6.25 Hz, *C*-5’), 57.36 (CH_2_*C*H Trp), 52.75 (*C*H_3_), 41.35 *(C*-2’), 31.02 (d, ^3^*J*_C-P_ = 7.5 Hz, *C*H_2_CH Trp). MS [ESI, m/z]: 739.12 [M+H], t_R_ = 14.65 min.

**(E)-5-(2-bromovinyl)-2’-deoxyuridine*-5’****-***(1-naphthyl(methoxy-L-tryptophanyl)-phosphate (35s)**

Purified by column chromatography (CH_2_Cl_2_/MeOH from 100/0 to 97/3), white solid, yield 14.3%.

^31^P NMR (202 MHz, MeOD-*d4*) δ 4.27. ^1^H-NMR (500 MHz; MeOD-*d4*) δ 8.04-8.01 (m, 1H, Ar*H*), 7.82-7.79 (m, 1H, Ar*H*), 7.66 (d, *J* = 7.8 Hz, 1H, Ar*H*), 7.53-7.48 (m, 4H, Ar*H),* 7.35-7.21 (m, 4H, *H*-5b, Ar*H*), 7.14-7.07 (m, 1H, Ar*H*), 7.03 (s, 1H, Ar*H*-2 Trp), 7.01-6.98 (m, 1H, Ar*H*), 6.61 (d, *J* = 13.4 Hz, 1H, *H*-5a), 6.12-6.10 (m, 1H, *H-*1’), 4.27-4.22 (m, 2H, C*H*CH_2_-Trp, *H*-3’), 4.14-4.19 (m, 1H, *H*-5’), 3.98-3.91 (m, 1H, *H*-4’), 3.91-3.82 (m, 1H, *H*-5’), 3.61 (s, 3H, C*H*_3_), 3.31-3.28 (m, 1H, CHC*H_2_*-Trp), 3.12-3.08 (m, 1H, CHC*H_2_* Trp), 2.13-2.08 (m, 1H, *H*-2’), 1.71-1.66 (m, 1H, *H*-2’). ^13^C-NMR (125 MHz; MeOD-*d4*), δ 175.12 (C=O ester), 163.52 (*C-*4), 150.89 (*C-*2), 147.87 (d, ^2^*J*_C-P_ = 6.25 Hz, ipso Nap), 139.33 (*C-*6), 138.07, 136.23, 130.41, 128.88, 128.56, 127.79, 127.71, 127.54, 126.47, 126.00, 124.99, 122.60, 122.46, 120.01, 119.18, 115.99, 115.97, 112.50, 112.37, 112.17, 110.74, (*C*-5, *C*-2 Nap, *C*-3 Nap, *C*-4 Nap, *C*-4a Nap, *C*-5 Nap, *C*-6 Nap, *C*-7 Nap, *C*-8 Nap, *C*-8a Nap, *C*-2 Trp, *C*-3 Trp, *C*-3a Trp, *C*-4 Trp, *C*-5 Trp, *C*-6 Trp, *C*-7 Trp, *C*-7a Trp, *C*-5a), 109.28 (*C*-5b), 86.97 (*C*-4’), 86.83 *(C*-1’), 72.15 *(C*-3’), 67.54 (d, ^2^*J*_C-P_= 5 Hz, *C*-5’), 57.44 (CH_2_*C*H Trp), 52.76 (*C*H_3_), 41.15 *(C*-2’), 31.03 (d, ^3^*J*_C-P_= 8.75 Hz, *C*H_2_CH Trp). MS [ESI, m/z]: 739.21 [M+H]. t_R_ = 14.71 min.

**(E)-5-(2-bromovinyl)-2’-deoxyuridine-***5’-***(1-naphthyl(ethoxy-L-tryptophanyl)-phosphate (36f)**

Purified by column chromatography (CH_2_Cl_2_/MeOH from 100/0 to 97/3), white solid, yield 13.2%.

^31^P NMR (202 MHz, MeOD) δ 4.58. ^1^H-NMR (500 MHz; MeOD) δ 8.12-8.08 (m, 1H, Ar*H*), 7.89-7.84 (m, 1H, Ar*H*), 7.65 (d, *J* = 7.6 Hz, 1H, Ar*H*), 7.58 (s, 1H*, H*-6), 7.54-7.42 (m, 3H, Ar*H),* 7.40-7.29 (m, 3H, Ar*H*), 7.23 (d, *J* = 13.2 Hz, 1H, *H*-5b), 7.11-7.09 (m, 2H, Ar*H*), 7.02-6.96 (m, 1H, Ar*H*), 6.64 (d, *J* = 13.2 Hz, 1H, *H*-5a), 6.11-6.08 (m, 1H, *H-*1’), 4.32-4.29 (m, 1H, C*H*CH_2_-Trp), 4.25-4.20 (m, 1H, *H*-3’), 4.08-4.01 (m, 3H, C*H_2_*CH_3_, *H*-5’), 3.97-3.91 (m, 1H, *H*-4’), 3.90-3.83 (m, 1H, CHC*H_2_*-Trp), 3.29-3.21 (m, 1H, CHC*H*_2_-Trp), 3.11-3.07 (m, 1H, CHC*H*_2_-Trp), 2.18-2.11 (m, 1H, *H*-2’), 1.73-1.67 (m, 1H, *H*-2’), 1.12-1.04 (m, 3H, C*H*_3_). ^13^C-NMR (125 MHz; MeOD), δ 174.52 (d, ^3^*J*_C-P_= 3.75 Hz, C=O ester), 163.53 (*C-*4), 150.88 (*C-*2), 147.69 (d, ^2^*J*_C-P_ = 6.25 Hz, ipso Nap), 139.38 (*C-*6), 137.98, 136.26, 130.41, 128.84, 128.62, 127.82, 127.52, 127.26, 126.67, 126.49, 125.53, 124.83, 122.64, 122.28, 119.72, 119.31, 116.51, 112.71, 112.36, 112.17, 110.71, (*C*-5, *C*-2 Nap, *C*-3 Nap, *C*-4 Nap, *C*-4a Nap, *C*-5 Nap, *C*-6 Nap, *C*-7 Nap, *C*-8 Nap, *C*-8a Nap, *C*-2 Trp, *C*-3 Trp, *C*-3a Trp, *C*-4 Trp, *C*-5 Trp, *C*-6 Trp, *C*-7 Trp, *C*-7a Trp, *C*-5a), 109.18 (*C*-5b), 87.14 (*C*-4’), 86.78 *(C*-1’), 72.23 *(C*-3’), 67.60 (d, ^2^*J*_C-P_= 5 Hz, *C*-5’), 63.68 (*C*H_2_ ester), 57.41 (CH_2_*C*H Trp), 41.34 *(C*-2’), 31.10 (d, ^3^*J*_C-P_= 7.5 Hz, *C*H_2_CH Trp), 14.33 (*C*H_3_). MS [ESI, m/z]: 753.48 [M+H]. t_R_ = 15.60 min.

**(E)-5-(2-bromovinyl)-2’-deoxyuridine-***5’-***(1-naphthyl(ethoxy-L-tryptophanyl)-phosphate (36s)**

Purified by column chromatography (CH_2_Cl_2_/MeOH from 100/0 to 97/3), white solid, 16.1% yield.

^31^P NMR (202 MHz, MeOD) δ 4.31. ^1^H-NMR (500 MHz; MeOD) δ 8.08-8.04 (m, 1H, Ar*H*), 7.88-7.82 (m, 1H, Ar*H*), 7.66 (d, *J* = 7.4 Hz, 1H, Ar*H*), 7.59-7.47 (m, 4H, *H*-6, Ar*H),* 7.37-7.23 (m, 4H, *H*-5b, Ar*H*), 7.12-7.09 (m, 1H, Ar*H*), 7.03 (s, 1H, *H-2 Trp*), 7.01-6.98 (m, 1H, Ar*H*), 6.63 (d, *J* = 13.6 Hz, 1H, *H*-5a), 6.12-6.09 (m, 1H, *H-*1’), 4.29-4.20 (m, 2H, 1H, C*H*CH_2_-Trp, *H*-3’), 4.16-4.12 (m, 1H, *H*-5’), 4.08-4.01 (m, 2H, C*H_2_*CH_3_), 3.95 (s, *H*-4’), 3.99-3.92 (m, 1H, *H*-5’), 3.31-3.25 (m, 1H, CHC*H*_2_-Trp), 3.12-3.08 (m, 1H, CHC*H*_2_-Trp), 2.12-2.09 (m, 1H, *H*-2’), 1.69-1.62 (m, 1H, *H*-2’), 1.16-1.10 (m, 3H, C*H*_3_). ^13^C-NMR (125 MHz; MeOD), δ 174.51 (C=O ester), 163.51 (*C-*4), 150.88 (*C-*2), 147.69 (d, ^2^*J*_C-P_ = 6.25 Hz, ipso Nap), 139.34 (*C-*6), 136.23, 130.46, 128.88, 128.61, 127.79, 127.51, 127.27, 126.71, 126.46, 126.00, 124.99, 122.55, 122.23, 119.98, 119.61, 119.30, 118.83, 116.01, 112.69, 112.47, 112.13, 110.82, (*C*-5, *C*-2 Nap, *C*-3 Nap, *C*-4 Nap, *C*-4a Nap, *C*-5 Nap, *C*-6 Nap, *C*-7 Nap, *C*-8 Nap, *C*-8a Nap, *C*-2 Trp, *C*-3 Trp, *C*-3a Trp, *C*-4 Trp, *C*-5 Trp, *C*-6 Trp, *C*-7 Trp, *C*-7a Trp, *C*-5a), 109.24 (*C*-5b), 86.92 (*C*-4’), 86.80 *(C*-1’), 72.16 *(C*-3’), 67.55 (d, ^2^*J*_C-P_= 5 Hz, *C*-5’), 62.45 (*C*H_2_ ester), 57.51 (CH_2_*C*H Trp), 41.14 *(C*-2’), 31.09 (d, ^3^*J*_C-P_= 8.75 Hz, *C*H_2_CH Trp), 14.47 (*C*H_3_). MS [ESI, m/z]: 753.46 [M+H]. t_R_ = 15.37 min.

**(E)-5-(2-bromovinyl)-2’-deoxyuridine-***5’-***(1-naphthyl(ethoxy-L-phenylalaninyl)-phosphate (37)**

Purified by column chromatography (CH_2_Cl_2_/MeOH from 100/0 to 97/3), white solid, yield 18 %.

^31^P NMR (202 MHz, MeOD) δ 4.58, 4.21. ^1^H-NMR (500 MHz; MeOD) δ 8.08-8.03 (m, 1H, Ar*H*), 7.89-7.83 (m, 1H, Ar*H*), 7.69-7.67 (m, 1H, Ar*H*), 7.60 (s, 1H, *H*-6), 7.56-7.50 (m, 2H, Ar*H),* 7.33- 7.28 (m, 3H, *H*-5b, Ar*H*), 7.23-7.16 (m, 5H, Ar*H)*, 6.70-6.65 (d, *J* = 13.6 Hz, 1H, *H*-5a), 6.20-6.18 (m, 1H, *H-*1’), 4.32-4.29 (m, 1H, *H*-3’), 4.25-4.20 (m, 1H, C*H*CH_2_), 4.18-4.01 (m, 4H, *H*-5’, C*H_2_*CH_3_), 3.99-3.91 (m, 1H, *H*-4’), 3.13-3.09 (m, 1H, CHC*H*_2_), 2.91-2.87 (m, 1H, CHC*H*_2_), 2.20-2.13 (m, 1H, *H*-2’), 1.84-1.87 (m, 1H, *H*-2’), 1.28-1.21 (m, 3H, C*H*_3_). ^13^C-NMR (125 MHz; MeOD), δ 174.21, 174.19 (C=O ester), 163.50 (*C-*4), 150.92 (*C-*2), 147.88, 147.83 (d, ^2^*J*_C-P_ = 6.25 Hz, ipso Nap), 139.47 (*C-*6), 138.25, 136.25, 130.71, 130.55, 129.61, 128.97, 128.92, 127.97, 127.72, 126.57, 126.25, 122.60, 116.57, 116.54, 116.01, 112.22 (*C*-5*, C*-2 Nap, *C*-3 Nap, *C*-4 Nap, *C*-4a Nap, *C*-5 Nap, *C*-6 Nap, *C*-7 Nap, *C*-8 Nap, *C*-8a Nap, ipso Ph, *C*-2 Ph, *C*-3 Ph, *C*-4 Ph, *C*-5 Ph, *C*-6 Ph, *C*-5a), 109.35 (*C*-5b), 87.13, 87.03 (*C*-4’), 86.86, 86.80 *(C*-1’), 72.28, 72.20 *(C*-3’), 67.67, 67.63 (*C*-5’), 62.43, 62.34 (*C*H_2_ ester), 58.18, 57.96 (CH_2_*C*H Trp), 40.95, 40.88 *(C*-2’), 31.61 (*C*H_2_CH Trp), 14.49 (*C*H_3_). MS [ESI, m/z]: 736.21 [M+Na], t_R_ = 16.09 min.

**(E)-5-(2-bromovinyl)-2’-deoxyuridine-***5’-***(1-naphthyl(methoxy-L-phenylalaninyl)-phosphate (38)**

Purified by column chromatography (CH_2_Cl_2_/MeOH from 100/0 to 97/3), white solid, yield 13.3 %. ^31^P NMR (202 MHz, MeOD) δ 4.45, 4.32. ^1^H-NMR (500 MHz; MeOD) δ 8.18-8.01 (m, 2H, Ar*H*), 7.88-7.81 (m, 2H, Ar*H*), 7.66-7.59 (m, 3H, *H*-5b, Ar*H*), 7.41-7.23 (m, 3H, Ar*H),* 7.23- 7.10 (m, 4H, Ar*H*), 6.68-6.63 (d, *J* = 13.6 Hz, 1H, *H*-5a), 6.20-6.11 (m, 1H, *H-*1’), 4.33-4.30 (m, 1H, *H*-3’), 4.31-4.20 (m, 3H, C*H*CH_2_, *H*-5’), 4.01-3.93 (m, 1H, *H*-4’), 3.12-3.02 (m, 1H, CHC*H*_2_), 2.91-2.78 (m, 1H, CHC*H*_2_), 2.21-2.13 (m, 1H, *H*-2’), 1.87-1.72 (m, 1H, *H*-2’), 1.23-1.20 (m, 3H, C*H*_3_).^13^C-NMR (125 MHz; MeOD), δ 174.36, 174.29 (C=O ester), 163.39 (*C-*4), 151.12 (*C-*2), 147.76, 147.68 (*C-*6), 139.51, 138.37, 136.28, 130.79, 130.65, 129.59, 128.94, 128.90, 127.96, 127.76, 126.58, 126.28, 122.57, 116.54, 116.21, 112.27 (*C*-5, ipso Nap*, C*-2 Nap, *C*-3 Nap, *C*-4 Nap, *C*-4a Nap, *C*-5 Nap, *C*-6 Nap, *C*-7 Nap, *C*-8 Nap, *C*-8a Nap, ipso Ph, *C*-2 Ph, *C*-3 Ph, *C*-4 Ph, *C*-5 Ph, *C*-6 Ph, *C*-5a), 109.39 (*C*-5b), 87.17, 87.08 (*C*-4’), 86.87, 86.82 *(C*-1’), 72.31, 72.22 *(C*-3’), 67.66, 67.61 (*C*-5’), 62.43, 62.34 (*C*H_3_), 58.21, 57.99 (CH_2_*C*H Trp), 40.91, 40.87 *(C*-2’), 32.01 (*C*H_2_CH Trp). MS [ESI, m/z]: 722.20 [M+Na]. t_R_ = 16.09 min.

**(E)-5-(2-bromovinyl)-2’-deoxyuridine-*5’-*(1-naphthyl(ethoxy-L-prolinyl)-phosphate (39)**

Purified by column chromatography (CH_2_Cl_2_/MeOH from 100/0 to 97/3), white solid, yield 17.3 %.

^31^P NMR (202 MHz, MeOD) δ 2.21. ^1^H-NMR (500 MHz; MeOD) δ 8.12-8.09 (m, 1H, Ar*H*), 7.91-7.87 (m, 1H, Ar*H*), 7.76-7.68 (m, 2H, *H*-6, Ar*H*), 7.58-7.51 (m, 2H, Ar*H*), 7.49-7.41 (m, 2H, Ar*H*), 7.32-7.29 (d, *J* = 13.8 Hz 1H, *H*-5b), 6.77-6.70 (d, *J* = 13.8 Hz 1H, *H*-5a), 6.21-6.18 (m, 1H, *H-*1’), 4.57-4.48 (m, 2H, *H*-5’) 4.42-4.43 (m, 2H, *H*-3’, C*H* Pro), 4.21-4.10 (m, 3H, *H*-4’, C*H_2_*CH_3_,), 3.49-3.43 (m, 1H, CHC*H*_2_ Pro), 3.38-3.32 (m, 1H, CHC*H*_2_ Pro), 2.26-2.19 (m, 2H, *H*-2’, C*H*_2_ Pro), 2.09-2.00 (m, 2H, C*H*_2_ Pro), 1.98-1.81 (m, 2H, *H*-2’, C*H*_2_ Pro), 1.30-1.23 (m, 3H, C*H*_3_). ^13^C-NMR (125 MHz; MeOD), δ 175.11 (C=O ester), 163.55 (*C-*4), 150.92 (*C-*2), 147.77 (d, ^2^*J*_C-P_ = 6.25 Hz, *ipso* Nap), 139.59 (*C-*6), 136.35, 130.53, 130.41, 130.25, 129.07, 128.01, 127.92, 127.74, 127.68, 126.55, 126.40, 126.28, 122.18, 115.78, 115.76, 112.16, 112.09 (*C*-5, *C*-2 Nap, *C*-3 Nap, *C*-4 Nap, *C*-4a Nap, *C*-5 Nap, *C*-6 Nap, *C*-7 Nap, *C*-8 Nap, *C*-8a Nap, *C*-5a,), 109.19 (*C*-5b), 87.35, 87.22 (*C*-4’), 86.90 *(C*-1’), 72.41 *(C*-3’), 68.29, 68.25 (*C*-5’), 62.56, 62.50, (CH_3_*C*H_2_), 61.95 (d, ^2^*J*_C-P_ = 6.25 Hz, *C*H Pro), 48.43, 48.39 (*C*H_2_CH Pro), 41.36, 41.21 *(C*-2’), 32.22, 32.14 (*C*H_2_ Pro), 26.20, 26.13 (*C*H_2_ Pro)14.59, 14.53 (*C*H_3_CH_2_). MS [ESI, m/z]: 686.09 [M+Na]. t_R_ = 15.08 min.

**(E)-5-(2-bromovinyl)-2’-deoxyuridine-*5’-*(1-naphthyl(ethoxy-L-O-Methyltyrosinyl)-phosphate (40)**

Purified by column chromatography (CH_2_Cl_2_/MeOH from 100/0 to 97/3), white solid, yield 18.7 %.

^31^P NMR (202 MHz, MeOD) δ 4.47, 4.21. ^1^H-NMR (500 MHz; MeOD) δ 8.12-8.04 (m, 1H, Ar*H*), 7.91-7.85 (m, 1H, Ar*H*), 7.70-7.60 (m, 2H, Ar*H*, *H*-6), 7.57-7.50 (m, 2H, Ar*H),* 7.39- 7.29 (m, 3H, *H*-5b, Ar*H*), 7.10-7.07 (m, 2H, Ar*H)*, 6.80-6.78 (m, 2H, Ar*H*)*,* 6.71-6.65 (m, 1H, *H*-5a), 6.21-6.12 (m, 1H, *H-*1’), 4.29-4.21 (m, 1H, *H*-3’), 4.19-4.00 (m, 6H, *H*-4’, *H*-5’, C*H*CH_2,_ C*H_2_*CH_3_), 3.74 (s, 3H, OCH_3_), 3.08-3.00 (m, 1H, CHC*H*_2_), 2.83-2.68 (m, 1H, CHC*H*_2_), 2.21-2.17 (m, 1H, *H*-2’), 1.87-1.78 (m, 1H, *H*-2’), 1.20-1.12 (m, 3H, C*H*_3_). ^13^C-NMR (125 MHz; MeOD), δ 174.08 (C=O ester), 163.54, 163.51 (*C-*4), 160.12 (*C*-OCH_3_, Tyr), 150.94 (*C-*2), 147.72 (*ipso* Nap), 139.50, 139.46 (*C-*6), 136.26, 131.56, 131.52, 131.23, 130.46, 130.43, 130.12, 130.03, 128.95, 128.91, 127.83, 127.80, 127.51, 127.17, 126.51, 126.17, 126.00, 122.66, 122.60, 116.43, 116.41, 115.98, 114.99, 114.97, 114.90, (*C*-2 Nap, *C*-3 Nap, *C*-4 Nap, *C*-4a Nap, *C*-5 Nap, *C*-6 Nap, *C*-7 Nap, *C*-8 Nap, *C*-8a Nap, *ipso* Ph, *C*-2 Ph, *C*-3 Ph, *C*-5 Ph, *C*-6 Ph, *C*-5a), 112.27, 112.22 (*C*-5), 109.34, 109.20 (*C*-5b), 87.11, 87.04, 86.87, 86.80, 86.73 *(C*-1’, *C*-4’), 72.25, 72.19 *(C*-3’), 67.72, 67.67, 67.64, 67.60 (*C*-5’), 62.49, 62.40 (*C*H_2_ ester), 58.35, 58.20 (CH_2_*C*H Tyr), 55.68, 55.66 (O*C*H_3_ Tyr), 41.33, 41.14 (*C*-2’), 40.18, 40.12 (*C*H_2_CH Tyr), 14.44, 14.41 (*C*H_3_). MS [ESI, m/z]: 766.12 [M+Na]. t_R_ = 14.77 min.

**(E)-5-(2-bromovinyl)-2’-deoxyuridine-5’-(1-naphthyl(pentyloxy-L-valinyl)-phosphate (41)**

Purified by column chromatography (CH_2_Cl_2_/MeOH from 100/0 to 97/3), white solid, yield 17.6%.

^31^P NMR (202 MHz, MeOD) δ 5.48, 5.34. ^1^H-NMR (500 MHz; MeOD) δ 8.28-8.13 (m, 2H, Ar*H*), 7.90-7.81 (m, 2H, Ar*H*), 7.71-7.69 (m, 1H, *H*-6), 7.61-7.43 (m, 4H, Ar*H*, *H*-5b), 6.75-6.68 (m, 1H, *H*-5a), 6.22-6.12 (m, 1H, *H-*1’), 4.47-4.30 (m, 3H, *H*-5’, *H*-3’), 4.18-4.10 (C*H*_2_ ester), 4.04-3.98 (m, 1H, *H*-4’), 3.82-3.68 (C*H*CO Val), 2.27-2.18 (m, 1H, *H*-2’), 1.98-1.86 (m, 2H, C*H*(CH_3_)_2_, *H*-2’), 1.71-1.52 (m, 2H, C*H*_2_), 1.49-1.31 (m, 4H, 2C*H*_2_), 1.01-0.83 (m, 9H, CH(C*H*_3_)_2,_ C*H*_3_).^13^C-NMR (125 MHz; MeOD), δ 173.01 (C=O ester), 163.72 (*C-*4), 151.26, 151.21 (*C-*2), 145.96 (ipso Nap), 139.60 (*C-*6), 136.32, 136.23, 130.44, 130.41, 128.97, 128.92, 128.60, 127.84, 127.54, 127.32, 126.71, 126.53, 126.20, 124.26, 123.39, 122.74, 122.70, 116.49, 115.30 (*C*-2 Nap, *C*-3 Nap, *C*-4 Nap, *C*-4a Nap, *C*-5 Nap, *C*-6 Nap, *C*-7 Nap, *C*-8 Nap, *C*-8a Nap, *C*-5a, *C*-5), 109.33, 109.24 (*C*-5b), 87.15 (*C*-4’), 86.78 *(C*-1’), 72.26, 72.16 *(C*-3’), 68.88, 68.07, 67.56 (*C*-5’), 66.39, 66.27, 66.19 (*C*H_2_), 62.18, 61.96 (NH*C*H Val), 41.27, 41.17 *(C*-2’), 33.66 ((*C*H_3_)_2_*C*H Val), 29.41, 29.37 (*C*H_2_), 23.28 (*C*H_2_), 19.58, 18.69 ( (*C*H_3_)_2_), 14.47, 14.37, 14.28 (*C*H_3_). MS [ESI, m/z]: 730.15 [M+Na]. t_R_ = 17.58 min.

**(E)-5-(2-bromovinyl)-2’-deoxyuridine-5’-(1-naphthyl(pentyloxy-L-phenylalaninyl)-phosphate (42)**

Purified by column chromatography (CH_2_Cl_2_/MeOH from 100/0 to 97/3), white solid, yield 12.9%.

^31^P NMR (202 MHz, MeOD) δ 4.53, 4.19. ^1^H-NMR (500 MHz; MeOD) δ 8.18-8.16 (m, 1H, Ar*H*), 7.91-7.88 (m, 2H, Ar*H*), 7.69-7.64 (m, 1H, Ar*H*)*,* 7.56-7.34 (m, 5H, Ar*H*), 7.30-7.21 (m, 5H, *H*-5b, *H*-6, Ar*H*), 6.87-6.79 (m, 1H, *H*-5a), 6.30-6.24 (m, 1H, *H-*1’), 4.46-4.41 (m, 1H, *H*-3’), 4.18-4.10 (m, 2H, *H*-5’), 4.12-4.08 (m, 2H, C*H_2_*O *n-Pnt*), 4.01-3.95 (m, 2H, *H*-4’, C*H*CH_2_ *Phe*), 3.14-3.09 (m, 1H, CHC*H*_2_), 2.91-2.83 (m, 1H, CHC*H*_2_), 2.29-2.19 (m, 1H, *H*-2’), 1.73-1.58 (m, 1H, *H*-2’), 1.26-1.20 (m, 6H, C*H*_2_), 0.90-0.75 (m, 3H, C*H*_3_). ^13^C-NMR (125 MHz; MeOD), δ 174.36 (C=O ester), 163.41 (*C-*4), 150.86 (*C-*2), 147.03, 146.98 (d, ^2^*J*_C-P_ = 6.25 Hz, *ipso* Naph), 138.84, 138.76, 138.43, 138.31, 136.18, 130.57, 130.43, 130.29, 129.58, 129.49, 129.39, 128.96, 128.87, 128.18, 127.94, 127.88, 127.78, 127.51, 126.36, 126.13, 121.69, 121.48, 118.90, 116.43, 116.10, 116.08 (*C-*6, *C*-5, *Ar* Phe, *C*-2 Nap, *C*-3 Nap, *C*-4 Nap, *C*-4a, Nap *C*-5 Nap, *C*-6 Nap, *C*-7 Nap, *C*-8 Nap, *C*-8a Nap, *C*-5a,), 110.60, 110.58 (*C*-5b), 87.59, 87.55 (*C*-4’), 87.22, 86.89, 86.81 *(C*-1’), 72.31, 72.26 (*C*-3’), 67.60 (d, ^2^*J*_C-P_ = 5 Hz *, C*-5’), 66.56, 66.47 (*C*H_2_), ), 58.27, 57.98 (CH_2_*C*H Phe), 41.47, 41.40 (*C*H_2_CH Phe), 41.11, 40.99 *(C*-2’), 29.31, 29.07 (*C*H_2_), 23.33 (*C*H_2_), 14.28, 14.22 (*C*H_3_). MS [ESI, m/z]: 778.14 [M+Na]. t_R_ = 17.92 min.

**(E)-5-(2-bromovinyl)-2’-deoxyuridine-5’-(1-naphthyl(ethoxy-L-tyrosinyl) phosphate (43)**

Purified by column chromatography (CH_2_Cl_2_/MeOH from 100/0 to 97/3), white solid, yield 14.6 %.

^31^P NMR (202 MHz, MeOD) δ 4.58, 4.28. ^1^H-NMR (500 MHz; MeOD) δ 8.12-8.04 (m, 1H, Ar*H*), 7.91-7.85 (m, 1H, Ar*H*), 7.70-7.61 (m, 2H, Ar*H*, *H*-6), 7.56-7.49 (m, 2H, Ar*H),* 7.37- 7.26 (m, 4H, *H*-5b, Ar*H*), 7.01-6.99 (m, 2H, Ar*H)*, 6.71-6.69 (m, 2H, Ar*H, H*-5a), 6.22-6.12 (m, 1H, *H-*1’), 4.33-4.28 (m, 1H, *H*-3’), 4.26-4.19 (m, 1H, C*H*CH_2_), 4.17-3.96 (m, 5H, *H*-4’, *H*-5’, C*H_2_*CH_3_), 3.03-2.98 (m, 1H, CHC*H*_2_), 2.81-2.71 (m, 1H, CHC*H*_2_), 2.20-2.13 (m, 1H, *H*-2’), 1.87-1.74 (m, 1H, *H*-2’), 1.22-1.12 (m, 3H, C*H*_3_). ^13^C-NMR (125 MHz; MeOD), δ 173.06 (C=O ester), 163.52 (*C-*4), 157.63, 157.60 (*C*-OH, Tyr), 150.92 (*C-*2), 147.89 (*ipso* Nap), 139.45, 139.41 (*C-*6), 136.26, 131.62, 131.57, 130.78, 130.64, 130.57, 129.60, 128.96, 128.91, 128.80, 128.67, 127.96, 127.83, 127.53, 126.55, 126.21, 126.03, 122.67, 122.57, 116.52, 116.47, 116.42, 116.06, 116.03, (*C*-2 Nap, *C*-3 Nap, *C*-4 Nap, *C*-4a Nap, *C*-5 Nap, *C*-6 Nap, *C*-7 Nap, *C*-8 Nap, *C*-8a Nap, *ipso* Ph, *C*-2 Ph, *C*-3 Ph, *C*-5 Ph, *C*-6 Ph, *C*-5a), 112.38, 112.31 (*C*-5), 109.32, 109.10 (*C*-5b), 87.03, 86.87, 86.81, 86.72 *(C*-1’, *C*-4’), 72.24, 72.23 *(C*-3’), 67.70, 67.65 (*C*-5’), 62.49, 62.39 (*C*H_2_ ester), 58.42, 58.38 (CH_2_*C*H Tyr), 41.45, 41.20 *(C*-2’), 40.28, 40.22, 40.18, 40.12 (*C*H_2_CH Tyr), 14.51, 14.43 (*C*H_3_). MS [ESI, m/z]: 752.21 [M+Na]. t_R_ = 13.32 min.

**(E)-5-(2-bromovinyl)-2’-deoxyuridine-5’-(1-naphthyl(ethoxy-L-methionyl) phosphate (44)**

Purified by column chromatography (CH_2_Cl_2_/MeOH from 100/0 to 97/3), white solid, yield 21.8 %.

^31^P NMR (202 MHz, MeOD) δ 4.95, 4.61. ^1^H-NMR (500 MHz; MeOD) δ 8.21-8.14 (m, 1H, Ar*H*), 7.92-7.88 (m, 1H, Ar*H*), 7.74-7.69 (m, 2H, *H*-6, Ar*H*), 7.59-7.50 (m, 3H, Ar*H*), 7.46-7.40 (m, 1H, Ar*H*), 7.39-7.30 (m, 1H, *H*-5b)*,* 6.81-6.70 (m, 1H*, H*-5a), 6.21-6.14 (m, 1H, *H-*1’), 4.49-4.31 (m, 3H, *H*-5’, *H*-3’), 4.20-4.07 (m, 4H, C*H*CH_2_, *H*-4’, C*H_2_*CH_3_), 2.50-2.37 (m, 2H, SC*H*_2_), 2.25-2.18 (m, 1H, *H*-2’), 2.10-1.98 (m, 1H, CHC*H*_2_ Met), 1.96-1.94 (m, 3H, C*H*_3_), 1.93-1.81 (m, 2H, *H*-2’, CHC*H*_2_ Met), 1.28-1.20 (m, 3H, C*H*_3_). ^13^C-NMR (125 MHz; MeOD), δ 174.65, 174.20 (C=O ester), 163.56 (*C-*4), 150.94 (*C-*2), 147.97 (*ipso* Nap), 139.64, 139.59 (*C-*6), 136.34, 130.52, 130.45, 128.99, 127.91, 127.62, 127.60, 126.53, 126.28, 122.67, 121.63, 116.50, 116.47, 112.24, 112.21 (*C*-2 Nap, *C*-3 Nap, *C*-4 Nap, *C*-4a, Nap *C*-5 Nap, *C*-6 Nap, *C*-7 Nap, *C*-8 Nap, *C*-8a Nap, *C*-5a, *C*-5), 109.29, 109.24 (*C*-5b), 87.24, 87.14, 87.07 (*C*-4’), 86.83, 86.77 (*C*-1’), 72.39, 72.17 *(C*-3’), 68.14, 68.09, 68.04, 68.00 (*C*-5’), 62.59, 61.56 (*C*H_2_ ester), 55.15, 55.03, 54.82 (CH_2_*C*H Met), 41.27, 41.22 *(C*-2’), 34.28, 34.23, 33.85, 33.78 (*C*H_2_CH Met), 30.97, 30.93 (*C*H_2_S), 15.17, 15.13 (*C*H_3_ Met), 14.50 (*C*H_3_ ester). MS [ESI, m/z]: 698.09 [M+H], 720.07 [M+Na]. t_R_ = 13.95 min.

**(E)-5-(2-bromovinyl)-2’-deoxyuridine-5’-(1-naphthyl(cyclohexyloxyglyinyl) phosphate (45)**

Purified by column chromatography (CH_2_Cl_2_/MeOH from 100/0 to 97/3), white solid, yield 22.1%.

^31^P NMR (202 MHz, MeOD) δ 5.88, 5.64. ^1^H-NMR (500 MHz, MeOD) δ 8.21-8.15 (m, 1H, Ar*H*), 7.95-7.88 (m, 2H, Ar*H, H*-6), 7.61-7.39 (m, 4H, Ar*H*), 7.34-7.28 (m, 1H, *H*-5b), 6.79-6.70 (m, 1H, *H*-5a), 6.27-6.17 (m, 1H, *H-*1’), 4.79-4.72 (m, 1H, *H*-1 cHex), 4.51-4.33 (m, 3H, *H*-5’, *H*-3’), 4.19-4.09 (m, 1H, *H*-4’), 3.89-3.72 (m, 2H, C*H*_2_), 2.27-2.18 (m, 1H, *H*-2’), 2.01-1.90 (m, 1H, *H*-2’), 1.89-1.70 (m, 4H, cHex), 1.60-1.51 (m, 1H, cHex), 1.49-1.23 (m, 5H, cHex). ^13^C-NMR (125 MHz; MeOD), δ 174.34, 174.11 (C=O ester), 164.21 (*C-*4), 150.56, 150.70 (*C-*2), 147.09 (*ipso* Nap), 139.58 (*C-*6), 136.35, 130.58, 130.53, 128.96, 127.86, 127.57, 126.53, 126.29, 126.21, 122.64, 122.57, 116.58 (*C*-2 Nap, *C*-3 Nap, *C*-4 Nap, *C*-4a, Nap *C*-5 Nap, *C*-6 Nap, *C*-7 Nap, *C*-8 Nap, *C*-8a Nap, *C*-5a, *C*-5), 109.03 (*C*-5b), 87.18, 87.07 (*C*-4’), 86.95, 86.88 *(C*-1’), 75.18 (*C*H-1 cHex), 72.30, 72.23 *(C*-3’), 68.00 (*C*-5’), 44.19 (*C*H_2_), 41.27, 41.14 *(C*-2’), 32.57, 26.39, 24.70 (*C*H_2_ cHex). MS [ESI, m/z]: 700.11 [M+Na]. t_R_ = 15.13 min.

**(E)-5-(2-bromovinyl)-2’-deoxyuridine-5’-(1-naphthyl(neopentyloxy-dimethylglyinyl)phosphate (46)**

Purified by column chromatography (CH_2_Cl_2_/MeOH from 100/0 to 97/3), white solid, yield 21.3%.

^31^P NMR (202 MHz, MeOD) δ 2.97, 2.87. ^1^H-NMR (500 MHz; MeOD) δ 8.23-8.18 (m, 1H, Ar*H*), 7.91-7.85 (m, 1H, Ar*H*)*,* 7.72-7.65 (m, 2H, *H*-6, Ar*H*), 7.58-7.49 (m, 3H, Ar*H*), 7.48-7.41 (m, 1H, Ar*H*)*,* 7.34-7.27 (m, 1H, *H*-5b), 6.79-6.64 (m, 1H, *H*-5a), 6.21-6.12 (m, 1H, *H-*1’), 4.47-4.33 (m, 3H, *H*-5’, *H*-3’, *H*-4’), 4.17-4.09 (m, 1H, *H*-5’), 3.82 (s, 2H, C*H*_2_), 2.23-2.17 (m, 1H, *H*-2’), 2.00-1.82 (m, 1H, *H*-2’), 1.61-1.49 (m, 6H, 2 C*H*_3_), 0.99-0.89 (m, 9H, 3 C*H*_3_). ^13^C-NMR (125 MHz; MeOD), δ 173.84 (C=O ester), 163.67 (*C-*4), 150.61 (*C-*2), 147.11 (*ipso* Nap), 139.84, 139.75 (*C-*6), 136.27, 130.54, 130.45, 128.91, 127.80, 127.41, 126.09, 122.94, 122.55, 116.58 (*C*-2 Nap, *C*-3 Nap, *C*-4 Nap, *C*-4a, Nap *C*-5 Nap, *C*-6 Nap, *C*-7 Nap, *C*-8 Nap, *C*-8a Nap, *C*-5a, *C*-5), 109.09 (*C*-5b), 87.13 (*C*-4’), 86.99 *(C*-1’), 75.83 (*c*), 72.23, 72.17 *(C*-3’), 68.07 (*C*-5’), 54.72 (*C*(CH_3_)_2_), 41.11, *(C*-2’), 32.31 (*C*(CH_3_)_3_), 27.63 (C(*C*H_3_)_2_), 26.81 (C(*C*H_3_)_3_). MS [ESI, m/z]: 716.14 [M+Na]. t_R_ = 16.63 min.

**(E)-5-(2-carboxymethoxyvinyl)-2’-deoxyuridine-5’-(1-naphthyl(ethoxy-L-tryptophanyl)-phosphate (47f)**

Purified by column chromatography (CH_2_Cl_2_/MeOH from 100/0 to 97/3), white solid, 10.1% yield.

^31^P NMR (202 MHz, MeOD) δ 4.55. ^1^H-NMR (500 MHz; MeOD) δ 8.09 (d, *J* = 8.2 Hz, 1H, Ar*H*), 7.87-7.83 (m, 2H, *H*-6, Ar*H*), 7.64 (d, *J* = 8.2 Hz, 1H, Ar*H*), 7.51-7.45 (m, 3H, Ar*H),* 7.41 (d, *J* = 7.8 Hz, 1H, Ar*H*), 7.32-7.29 (m, 2H, Ar*H*)*,* 7.27 (d, *J* = 13.5 Hz 1H, *H*-5b), 7.08-7.06 (m, 2H, Ar*H*), 6.95-6.92 (m, 1H, Ar*H*), 6.82 (d, *J* = 13.6 Hz, 1H, *H*-5a), 6.11-6.09 (m, 1H, *H-*1’), 4.45-4.40 (m, 1H, C*H*CH_2_), 4.24-4.20 (m, 1H, *H*-3’), 4.15-3.95 (m, 5H, *H*-5’, C*H_2_*CH_3_, *H*-4’), 3.95-3.90 (m, 1H, CHC*H_2_*), 3.63 (s, 3H, C*H*_3_CO), 3.27-3.20 (m, 1H, CHC*H_2_*), 3.12-3.08 (m, 1H, CHC*H_2_*), 2.20-2.18 (m, 1H, *H*-2’), 1.82-1.76 (m, 1H, *H*-2’), 1.11-1.08 (m, 3H, C*H*_3_). ^13^C-NMR (125 MHz; MeOD), δ 174.52 (C=O ester), 169.56 (*C*OC=C), 163.42 (*C-*4), 150.81 (*C-*2), 147.75 (d, ^2^*J*_C-P_ = 7.5 Hz, ipso Nap), 143.99 (*C-*6), 138.88, 138.12, 137.96, 136.20, 128.88, 128.78, 127.78, 127.44, 126.47, 126.09, 124.87, 122.68, 122.49, 119.92, 119.26, 118.76, 116.45, 112.38, 110.74, 110.53 (*C*-5, *C*-2 Nap, *C*-3 Nap, *C*-4 Nap, *C*-4a Nap, *C*-5 Nap, *C*-6 Nap, *C*-7 Nap, *C*-8 Nap, *C*-8a Nap, *C*-2 Trp, *C*-3 Trp, *C*-3a Trp, *C*-4 Trp, *C*-5 Trp, *C*-6 Trp, *C*-7 Trp, *C*-7a Trp, *C*-5a, *C*-5b), 87.52 *(C*-1’), 86.92 (d, ^2^*J*_C-P_= 8.75 Hz, *C*-4’), 72.14 *(C*-3’), 67.58 (d, ^2^*J*_C-P_= 5 Hz, *C*-5’), 62.83 (*C*H_2_ ester), 57.40 (CH_2_*C*H Trp), 51.95 (*C*H_3_OCO), 41.45 *(C*-2’), 31.10 (d, ^3^*J*_C-P_= 7.5 Hz, *C*H_2_CH Trp), 14.49 (*C*H_3_). MS [ESI, m/z]: 755.32 [M+Na]. t_R_ = 13.77 min.

**(E)-5-(2-carboxymethoxyvinyl)-2’-deoxyuridine-5’-(1-naphthyl(ethoxy-L-tryptophanyl)-phosphate (47s)**

Purified by column chromatography (CH_2_Cl_2_/MeOH from 100/0 to 97/3), white solid, 10.9% yield.

^31^P NMR (202 MHz, MeOD) δ 4.30. ^1^H-NMR (500 MHz; MeOD) δ 8.00 (d, *J* = 7.9 Hz, 1H, Ar*H*), 7.8 (d, *J* = 8.2 Hz, 1H, Ar*H*), 7.72 (s, 1H, *H*-6), 7.62-7.55 (m, 2H, Ar*H*), 7.49-7.42 (m, 3H, Ar*H),* 7.38-7.22 (m, 4H, *H*-5b, Ar*H*)*,* 7.12-7.06 (m, 2H, Ar*H*), 7.01-6.95 (m, 1H, Ar*H*), 6.90 (d, *J* = 13.4 Hz, 1H, *H*-5a), 6.03-6.00 (m, 1H, *H-*1’), 4.28-4.18 (m, 2H, C*H*CH_2_, *H*-3’), 4.13-4.05 (m, 2H, C*H_2_*CH_3_), 4.01-3.92 (m, 3H, *H*-5’, *H*-4’), 3.73-3.68 (m, 1H, *H*-5’), 3.64 (s, 3H, C*H*_3_CO), 3.31-3.23 (m, 1H, CHC*H_2_*), 3.12-3.07 (m, 1H, CHC*H_2_*), 2.13-2.09 (m, 1H, *H*-2’), 1.60-1.52 (m, 1H, *H*-2’), 1.09-1.03 (m, 3H, C*H*_3_). ^13^C-NMR (125 MHz; MeOD), δ 174.66 (d, ^3^*J*_C-P_= 2.5 Hz, C=O ester), 169.64 (*C*OC=C), 163.40 (*C-*4), 150.81 (*C-*2), 147.92 (d, ^2^*J*_C-P_ = 6.25 Hz, *ipso* Nap), 143.90 (*C-*6), 139.01, 138.17, 138.02, 137.94, 136.15, 128.71, 127.82, 127.67, 127.49, 126.48, 126.11, 125.30, 125.13, 124.88, 122.51, 119.96, 119.29, 118.83, 116.44, 115.97, 112.45, 110.94, 110.50 (*C*-5, *C*-2 Nap, *C*-3 Nap, *C*-4 Nap, *C*-4a Nap, *C*-5 Nap, *C*-6 Nap, *C*-7 Nap, *C*-8 Nap, *C*-8a Nap, *C*-2 Trp, *C*-3 Trp, *C*-3a Trp, *C*-4 Trp, *C*-5 Trp, *C*-6 Trp, *C*-7 Trp, *C*-7a Trp, *C*-5a, *C*-5b), 87.59 *(C*-1’), 87.18 (*C*-4’), 72.24 *(C*-3’), 67.47 (d, ^2^*J*_C-P_= 5 Hz, *C*-5’), 62.45 (*C*H_2_ ester), 57.50 (CH_2_*C*H Trp), 52.07 (*C*H_3_OCO), 41.39 *(C*-2’), 31.03 (d, ^3^*J*_C-P_= 7.5 Hz, *C*H_2_CH Trp), 14.53 (*C*H_3_). MS [ESI, m/z]: 755.31 [M+Na]. t_R_ = 13.87 min.

**(E)-5-(2-carboxymethoxyvinyl)-2’-deoxyuridine-5’-(1-naphthyl(ethoxy-L-valinyl)-phosphate (48)**

Purified by column chromatography (CH_2_Cl_2_/MeOH from 100/0 to 97/3), white solid, yield 16.2 %.

^31^P NMR (202 MHz, MeOD) δ 5.44, 5.22. ^1^H-NMR (500 MHz; MeOD) δ 8.18-8.12 (m, 1H, Ar*H*), 8.01-7.96 (m, 1H, Ar*H*), 7.86-7.81 (s, 1H, *H*-6), 7.65-7.60 (m, 1H, Ar*H*), 7.58-7.43 (m, 3H, Ar*H),* 7.41-7.29 (m, 2H, *H*-5b, Ar*H*), 6.91 -6.88 (m, 1H, *H*-5a), 6.22-6.16 (m, 1H, *H-*1’), 4.50-4.41 (m, 2H, *H*-5’, *H*-3’), 4.22-4.15 (m, 1H, *H*-5’), 4.12-4.03 (m, 3H, *H*-4’, C*H_2_*CH_3_), 3.82-3.71 (m, 1H, C*H*CO), 3.67 (s, 3H, C*H*_3_CO), 2.32-2.24 (m, 1H, *H*-2’), 2.12-1.91 (m, 2H, *H*-2’, C*H*(CH_3_)_2_), 1.24-1.20 (m, 3H, C*H*_3_), 0.91-.82 (m, 6H, C*H*(CH_3_)_2_) .^13^C-NMR (125 MHz; MeOD), δ 174.75 (d, ^3^*J*_C-P_= 2.5 Hz, C=O ester), 169.70 (*C*OC=C), 163.38 (*C-*4), 151.21 (*C-*2), 147.87 (d, ^2^*J*_C-P_=6.25 Hz, *ipso* Nap), 143.87 (*C-*6), 139.21, 138.19, 138.08, 137.91, 136.21, 128.73, 127.77, 127.63, 127.51, 126.46, 126.17, 125.31, 125.15, 124.76, 122.53,119.99, 119.31, 118.81, 116.43, 115.95, 112.42, 110.91, 110.43 (*C*-5, *C*-2 Nap, *C*-3 Nap, *C*-4 Nap, *C*-4a Nap, *C*-5 Nap, *C*-6 Nap, *C*-7 Nap, *C*-8 Nap, *C*-8a Nap, *C*-5a, *C*-5b), 87.17 (d, ^2^*J*_C-P_= 6.25 Hz, *C*-4’), 86.58 *(C*-1’), 72.23 *(C*-3’), 67.46 (d, ^2^*J*_C-P_= 5 Hz, *C*-5’), 62.45 (NH*C*H Val), 52.06 (CH_3_OCO), 41.38 *(C*-2’), 32.53 (d, ^3^*J*_C-P_= 7.5 Hz, (*C*H_3_)_2_*C*H Val), 19.76, 19.65 ( (*C*H_3_)_2_). MS [ESI, m/z]: 646.32 [M+H], 668.29 [M+Na]. t_R_ = 13.63 min.

**(E)-5-(2-carboxymethoxyvinyl)-2’-deoxyuridine-5’-(1-naphthyl(benzyloxy-L-alanine)-phosphate (49f)**

Purified by column chromatography (CH_2_Cl_2_/MeOH from 100/0 to 97/3), white solid, yield 12.2%. ^31^P NMR (202 MHz, MeOD) δ 4.31. ^1^H-NMR (500 MHz; MeOD) δ 8.13 (d, *J* = 8.2 Hz, 1H, Ar*H*), 7.95 (s, 1H, *H*-6), 7.87 (d, *J* = 8.2 Hz, 1H, Ar*H*), 7.68 (d, *J* = 8.4 Hz, 1H, Ar*H*), 7.53-7.48 (m, 3H, Ar*H)*, 7.38-7.29 (m, 7H, Ar*H*, *H*-5b), 6.86 (d, *J* = 13.6 Hz, 1H, *H*-5a), 6.13-6.10 (m, 1H, *H-*1’), 5.12 (s, 2H, C*H*_2_Ph), 4.42-4.38 (m, 1H, *H*-5’), 4.37- 4.32 (m, 1H, *H*-3’), 4.30-4.25 (m, 1H, *H*-5’), 4.16-4.09 (m, 2H, *H*-4’, C*H*CH_3_), 3.64 (s, 3H, C*H_3_*CO), 2.26-2.10 (m, 1H, *H*-2’), 1.89-1.81 (m, 1H, *H*-2’), 1.39-1.33 (m, 3H, C*H*_3_). ^13^C-NMR (125 MHz; MeOD), δ 174.59 (d, ^3^*J*_C-P_= 5 Hz, C=O ester), 169.51 (*C*OC=C), 163.42 (*C-*4), 150.80 (*C-*2), 147.80 (d, ^2^*J*_C-P_ = 7.5 Hz, *ipso* Nap), 144.15 (*C-*6), 138.88, 137.15, 136.24, 129.61, 129.58, 129.38, 129.33, 129.12, 128.95, 128.08, 127.85, 127.81, 127.54, 127.19, 126.51, 126.18, 122.62, 118.86, 116.58, 116.55 (*C*-2 Nap, *C*-3 Nap, *C*-4 Nap, *C*-4a Nap, *C*-5 Nap, *C*-6 Nap, *C*-7 Nap, *C*-8 Nap, *C*-8a Nap, ipso Ph, *C*-2 Ph, *C*-3 Ph, *C*-4 Ph, *C*-5 Ph, *C*-6 Ph, *C*-5a, *C*-5), 110.54 (*C*-5b), 87.55 *(C*-1’), 86.98 (d, ^2^*J*_C-P_= 7.5 Hz, *C*-4’), 72.09 *(C*-3’), 68.07 (Ph*C*H_2_ ester), 67.82 (d, ^2^*J*_C-P_= 5 Hz, *C*-5’), 51.96 (*C*H_3_CO), 51.86 (CH_3_*C*H Ala) 41.43 *(C*-2’), 20.46 (d, ^3^*J*_C-P_= 6.25 Hz, *C*H_3_). (fm) MS [ESI, m/z]: 702.18 [M+Na]. t_R_ = 13.18 min.

**(E)-5-(2-carboxymethoxyvinyl)-2’-deoxyuridine-5’-(1-naphthyl(benzyloxy-L-alanine)-phosphate (49s)**

Purified by column chromatography (CH_2_Cl_2_/MeOH from 100/0 to 97/3), white solid, yield 13.1%.

^31^P NMR (202 MHz, MeOD) δ 4.64. ^1^H-NMR (500 MHz; MeOD) δ 8.11 (d, *J* = 8.3 Hz, 1H, Ar*H*), 8.00 (s, 1H, *H*-6), 7.86 (d, *J* = 8.3 Hz, 1H, Ar*H*), 7.69 (d, *J* = 8.4 Hz, 1H, Ar*H*), 7.54-7.38 (m, 4H, Ar*H)*, 7.37-7.28 (m, 6H, Ar*H*, *H*-5b), 6.88 (d, *J* = 13.6 Hz, 1H, *H*-5a), 6.19-6.16 (m, 1H, *H-*1’), 5.06 (dd, *J* = 27.9, 12.3 Hz, 2H, C*H*_2_Ph), 4.43-4.39 (m, 2H, *H*-5’), 4.39- 4.35 (m, 1H, *H*-3’), 4.18-4.10 (m, 2H, *H*-4’, C*H*CH_3_), 3.62 (s, 3H, C*H_3_*CO), 2.25-2.11 (m, 1H, *H*-2’), 1.90-1.83 (m, 1H, *H*-2’), 1.39-1.34 (m, 3H, C*H*_3_). ^13^C-NMR (125 MHz; MeOD), δ 174.93 (d, ^3^*J*_C-P_= 3.75 Hz, C=O ester), 169.58 (*C*OC=C), 163.45 (*C-*4), 150.84 (*C-*2), 147.93 (ipso Nap), 144.21 (*C-*6), 138.94, 137.15, 136.26, 129.57, 129.33, 129.32, 128.88, 128.08, 127.85, 127.51, 126.48, 126.15, 122.65, 118.85, 116.36, 116.34 (*C*-2 Nap, *C*-3 Nap, *C*-4 Nap, *C*-4a Nap, *C*-5 Nap, *C*-6 Nap, *C*-7 Nap, *C*-8 Nap, *C*-8a Nap, ipso Ph, *C*-2 Ph, *C*-3 Ph, *C*-4 Ph, *C*-5 Ph, *C*-6 Ph, *C*-5a, *C*-5), 110.56 (*C*-5b), 87.56 *(C*-1’), 87.20 (d, ^2^*J*_C-P_= 7.5 Hz, *C*-4’), 72.21 *(C*-3’), 68.07 (Ph*C*H_2_ ester), 67.95 (d, ^2^*J*_C-P_= 5 Hz, *C*-5’), 54.82 (*C*H_3_CO), 51.95 (CH_3_*C*H Ala) 41.40 *(C*-2’), 20.30 (d, ^3^*J*_C-P_= 8.75 Hz, *C*H_3_). MS [ESI, m/z]: 680.19 [M+H], 702.18 [M+Na], t_R_ = 13.40 min.

**(E)-5-(2-carboxymethoxyvinyl)-2’-deoxyuridine-5’-(1-naphthyl(cyclohexyloxy-L-valinyl)-phosphate (50)**

Purified by column chromatography (CH_2_Cl_2_/MeOH from 100/0 to 97/3), white solid, yield 17.9%.

^31^P NMR (202 MHz, MeOD) δ 5.52, 5.30. ^1^H-NMR (500 MHz; MeOD) δ 8.18-8.12 (m, 1H, Ar*H*), 8.00-7.98 (m, 1H, *H*-6), 7.88-7.83 (m, 1H, Ar*H*), 7.69-7.64 (m, 1H, Ar*H*), 7.55-7.44 (m, 3H, Ar*H)*, 7.41-7.31 (m, 2H, Ar*H*, *H*-5b), 6.92-6.84 (m, 1H, *H*-5a), 6.18-6.12 (m, 1H, *H-*1’), 4.72-4.62 (m, 1H, *H*-1 cHex), 4.49-4.32 (m, 3H, *H*-5’, *H*-3’), 4.19-4.12 (m, 1H, *H*-4’), 3.81-3.72 (C*H*CO Val), 3.68 (s, 3H, C*H_3_*CO), 2.31-2.21 (m, 1H, *H*-2’), 2.12-2.06 (m, 1H, C*H*(CH_3_)_2_), 1.95-1.86 (m, 1H, *H*-2’), 1.79-1.62 (m, 3H, cHex), 1.54-1.47 (m, 1H, cHex), 1.44-1.29 (m, 6H, cHex), 0.94-0.85 (m, 6H, CH(C*H*_3_)_2_). ^13^C-NMR (125 MHz; MeOD), δ 173.74, 173.49 (C=O ester), 169.56, 169.52 (*C*OC=C), 163.42 (*C-*4), 150.81 (*C-*2), 148.05, 147.99, 147.88, 147.82 (ipso Nap), 144.28, 144.20 (*C-*6), 138.88, 138.41, 136.35, 136.25, 135.16, 129.05, 129.02, 128.94, 128.89, 128.08, 127.88, 127.79, 127.47, 127.08, 126.51, 126.48, 126.11, 122.74, 122.34, 118.78, 118.72, 116.71, 116.48, 116.41, 116.39 (*C*-2 Nap, *C*-3 Nap, *C*-4 Nap, *C*-4a Nap, *C*-5 Nap, *C*-6 Nap, *C*-7 Nap, *C*-8 Nap, *C*-8a Nap, *C*-5a, *C*-5), 110.66, 110.60 (*C*-5b), 87.38, 87.24, 87.18, 87.03 (*C*-4’), 86.93, 86.55 *(C*-1’), 75.00, 74.96 (*C*H-1 cHex), 72.22, 72.12, 71.95 *(C*-3’), 68.08, 68.04, 68.00 (*C*-5’), 62.17, 62.11, 62.00 (NH*C*H Val), 51.94 (*C*H_3_CO), 41.37, 41.33 *(C*-2’), 33.35, 33.30, 33.06, 33.00 ((*C*H_3_)_2_*C*H Val), 32.55, 32.51, 32.48, 26.38, 24.62 (*C*H_2_ cHex), 19.86, 19.57 ( (*C*H_3_)_2_). MS [ESI, m/z]: 700.26 [M+H], 722.24 [M+Na]. t_R_ = 15.66 min.

**(E)-5-(2-carboxymethoxyvinyl)-2’-deoxyuridine-5’-(1-naphthyl(ethoxy-L-prolinyl)-phosphate (51)**

Purified by column chromatography (CH_2_Cl_2_/MeOH from 100/0 to 97/3), white solid, yield 19.7 %.

^31^P NMR (202 MHz, MeOD) δ 2.40. ^1^H-NMR (500 MHz; MeOD) δ 8.10-8.05 (m, 2H, *H*-6, Ar*H*), 7.91-7.88 (m, 1H, Ar*H*), 7.73-7.70 (m, 1H, Ar*H*), 7.58-7.42 (m, 4H, Ar*H*), 7.41-7.35 (d, *J* = 13.7 Hz 1H, *H*-5b), 6.94-6.89 (d, *J* = 13.7 Hz 1H, *H*-5a), 6.18-6.12 (m, 1H, *H-*1’), 4.59-4.50 (m, 2H, *H*-5’), 4.47- 4.41 (m, 1H, *H*-3’), 4.38-4.32 (m, 1H, C*H* Pro), 4.22-4.10 (m, 3H, *H*-4’, C*H_2_*CH_3_), 3.67 (s, 3H, C*H*_3_), 3.42-3.39 (m, 1H, C*H*_2_ Pro), 2.24-2.18 (m, 2H, CHC*H*_2_ Pro, *H*-2’), 2.11-2.02 (m, 2H, C*H*_2_ Pro), 1.99-1.91 (m, 1H, C*H*_2_ Pro), 1.89-1.81 (m, 1H, C*H*_2_ Pro), 1.79-1.71 (m, 1H, *H*-2’), 1.29-1.24 (m, 3H, C*H*_3_). ^13^C-NMR (125 MHz; MeOD), δ 175.12 (C=O ester), 169.48 (*C*OC=C), 163.54 (*C-*4), 150.91 (*C-*2), 148.73 (ipso Nap), 144.16 (*C-*6), 138.96, 136.32, 129.02, 127.98, 127.67, 126.50, 126.18, 122.13, 118.81, 115.78, 115.72, 112.09 (*C*-5, *C*-2 Nap, *C*-3 Nap, *C*-4 Nap, *C*-4a Nap, *C*-5 Nap, *C*-6 Nap, *C*-7 Nap, *C*-8 Nap, *C*-8a Nap, *C*-5a), 110.40 (*C*-5b), 87.78 (*C*-4’), 87.58, 87.52 *(C*-1’), 72.51 *(C*-3’), 68.29, 68.25 (*C*-5’), 62.51, 62.50, (CH_3_*C*H_2_), 62.46 (*C*H Pro), 51.92 (*C*H_3_OCO), 48.43, 48.39 (*C*H_2_CH Pro), 41.60 *(C*-2’), 32.16, 32.09 (*C*H_2_ Pro), 26.24, 26.16 (*C*H_2_ Pro), 14.44 (*C*H_3_CH_2_). MS [ESI, m/z]: 644.20 [M+H], 666.18 [M+Na]. t_R_ = 12.47 min

**(E)-5-(2-carboxymethoxyvinyl)-2’-deoxyuridine-5’-(1-naphthyl(pentoxy-L-phenylalaninyl) phosphate (52)**

Purified by column chromatography (CH_2_Cl_2_/MeOH from 100/0 to 97/3), white solid, yield 18.9 %.

^31^P NMR (202 MHz, MeOD) δ 4.47, 4.17. ^1^H-NMR (500 MHz; MeOD) δ 8.11-8.02 (m, 1H, Ar*H*), 7.92-7.84 (m, 2H, Ar*H*), 7.68-7.61 (m, 1H, Ar*H*)*,* 7.51-7.32 (m, 5H, Ar*H*), 7.28-7.17 (m, 5H, *H*-5b, *H*-6, Ar*H*), 6.93-6.84 (m, 1H, *H*-5a), 6.12-6.08 (m, 1H, *H-*1’), 4.30-4.24 (m, 1H, *H*-3’), 4.21-4.15 (m, 2H, *H*-5’), 4.12-4.08 (m, 2H, C*H_2_*O *n-Pnt*), 4.02-3.92 (m, 2H, *H*-4’, C*H*CH_2_ *Phe*), 3.68 (s, 3H, C*H*_3_), 3.14-3.09 (m, 1H, CHC*H*_2_), 2.91-2.83 (m, 1H, CHC*H*_2_), 2.28-2.21 (m, 1H, *H*-2’), 1.53-1.46 (m, 1H, *H*-2’), 1.32-1.18 (m, 6H, C*H*_2_), 0.88-0.61 (m, 3H, C*H*_3_). ^13^C-NMR (125 MHz; MeOD), δ 174.56 (C=O ester), 169.60, 169.54 (*C*OC=C), 163.41 (*C-*4), 150.81, 150.75 (*C-*2), 144.09, 143.98 (*ipso* OPh), 138.94, 138.91, 138.44, 138.19, 136.21, 130.68, 130.55, 130.21, 129.62, 129.58, 129.51, 128.92, 128.89, 128.00, 127.96, 127.82, 127.49, 127.45, 126.53, 126.14, 121.73, 121.57, 118.89, 116.47, 116.03, 116.01 (*C-*6, *C*-5, *Ar* Phe, *C*-2 Nap, *C*-3 Nap, *C*-4 Nap, *C*-4a, Nap *C*-5 Nap, *C*-6 Nap, *C*-7 Nap, *C*-8 Nap, *C*-8a Nap, *C*-5a,), 110.55, 110.51 (*C*-5b), 87.63, 87.56, 87.24 (*C*-4’), 87.19, 86.95, 86.89 *(C*-1’), 72.25, 72.16 (*C*-3’), 67.59 (d, ^2^*J*_C-P_ = 5 Hz, *C*-5’), 66.61, 66.54, 66.41 (*C*H_2_), 58.26, 57.96 (CH_2_*C*H Phe), 52.03, 51.97 (*C*H_3_OCO), 41.51, 41.41 (*C*H_2_CH), 41.10, 40.99 *(C*-2’), 29.29, 29.08 (*C*H_2_), 23.32 (*C*H_2_), 14.27, 14.25 (*C*H_3_). MS [ESI, m/z]: 736.26 [M+H], 758.24 [M+Na]. t_R_ = 16.40 min.

**(E)-5-(2-carboxymethoxyvinyl)-2’-deoxyuridine-5’-(1-naphthyl(ethoxy-L-methionyl)phosphate (53)**

Purified by column chromatography (CH_2_Cl_2_/MeOH from 100/0 to 97/3), white solid, yield 18.3 %.

^31^P NMR (202 MHz, MeOD) δ 4.99, 4.60. ^1^H-NMR (500 MHz; MeOD) δ 8.21-8.12 (m, 1H, Ar*H*), 8.07-8.01 (m, 1H, *H*-6), 7.91-7.85 (m, 1H, Ar*H*), 7.73-7.67 (m, 1H, Ar*H*), 7.56-7.31 (m, 5H, Ar*H*, *H*-5b)*,* 6.95-6.87 (m, 1H*, H*-5a), 6.19-6.13 (m, 1H, *H-*1’), 4.53-4.33 (m, 3H, *H*-5’, *H*-3’), 4.23-4.02 (m, 4H, C*H*CH_2_, *H*-4’, C*H_2_*CH_3_), 3.69 (s, 3H, C*H*_3_), 2.50-2.36 (m, 2H, SC*H*_2_), 2.30-2.19 (m, 2H, *H*-2’), 2.19-2.01 (m, 1H, CHC*H*_2_ Met), 1.96-1.91 (m, 3H, C*H*_3_), 1.88-1.80 (m, 1H, CHC*H*_2_ Met), 1.24-1.17 (m, 3H, C*H*_3_). ^13^C-NMR (125MHz; MeOD), δ 174.77 (C=O ester), 169.56 (*C*OC=C), 163.75 (*C-*4), 151.84 (*C-*2), 147.97 (*ipso* Nap), 144.25 (*C-*6), 139.02, 138.92, 136.29, 128.93, 127.87, 127.54, 127.51, 126.50, 126.18, 122.71, 122.63, 118.86, 116.43 (*C*-2 Nap, *C*-3 Nap, *C*-4 Nap, *C*-4a, Nap *C*-5 Nap, *C*-6 Nap, *C*-7 Nap, *C*-8 Nap, *C*-8a Nap, *C*-5a, *C*-5), 110.61, 110.56 (*C*-5b), 87.69, 87.52, 87.39, 87.33 (*C*-4’), 86.98, 86.92 (*C*-1’), 72.36, 72.06 *(C*-3’), 68.07, 67.95, 67.90 (*C*-5’), 62.56, 61.51 (*C*H_2_ ester), 55.18, 55.08 (CH_2_*C*H Met), 51.93 (*C*H_3_OCO), 41.44, 41.40 *(C*-2’), 34.26, 34.20, 33.75 (*C*H_2_CH Met), 30.95, 30.93 (*C*H_2_S), 15.11, 15.09 (*C*H_3_ Met), 14.45, 14.41 (*C*H_3_ ester). MS [ESI, m/z]: 678.18 [M+H], 700.16 [M+Na], t_R_ = 12.56 min

**S2** **Cytostatic Activity Assays**

The tumor cells were seeded in 96-well microtiter plates and exposed to different concentrations of the test compounds. After 2 days (L1210, L1210/TK^-^) and 3 days (CEM, CEM/TK^-^, HeLa, HeLa/TK^-^), cell number was determined using a Particle counter (Coulter Z-1, Analis, Ghent, Belgium). The IC_50_ represents the compound concentration required to inhibit tumor cell proliferation by 50%.

**S3 Enzymatic Procedure: Carboxypeptidase Y assay**

The enzymatic activation of the protides towards carboxypeptidase Y was studied using in situ ^31^P NMR. The experiment was carried out by dissolving either **35f** or **35s** (5.0 mg) in d_6_-acetone (0.15 mL) and adding Trizma buffer pH 7.6 (0.30 mL). The resulting solution was placed in an NMR tube and a ^31^P NMR experiment at 25 ^o^C was recorded as the blank experiment. The enzyme carboxypeptidase Y (0.1 mg) was dissolved in Trizma (0.15 mL) and added to the solution of the phosphoramidate derivative in the NMR tube. ^31^P-NMR experiment was performed recording the experiment every 7 min at 25 ^o^C.

**S4 Computational study**

Molecular modelling studies were performed on a MacPro dual 2.66GHz Xeon running Ubuntu 8 using Molecular Operating Environment (MOE) 2008.10. Hydrogen atoms were added to the crystal structure (PDB; 1YSC) ^25^ and minimised with MOE until a gradient of 0.05 Kcal mol^-1^ Å^-1^ was reached, using the MMFF94x forcefield. The partial charges were automatically calculated. Docking experiments were carried out using the MOE GUI of FlexX implemented in MOE.

The suggested mechanism of action of cathepsin involves an attack from the side chain OH group of residue Ser 146 to the ester carbonyl C=O group of the ProTide, which is also hydrogen-bonded to the backbone NH group of residue Gly 52^26^.


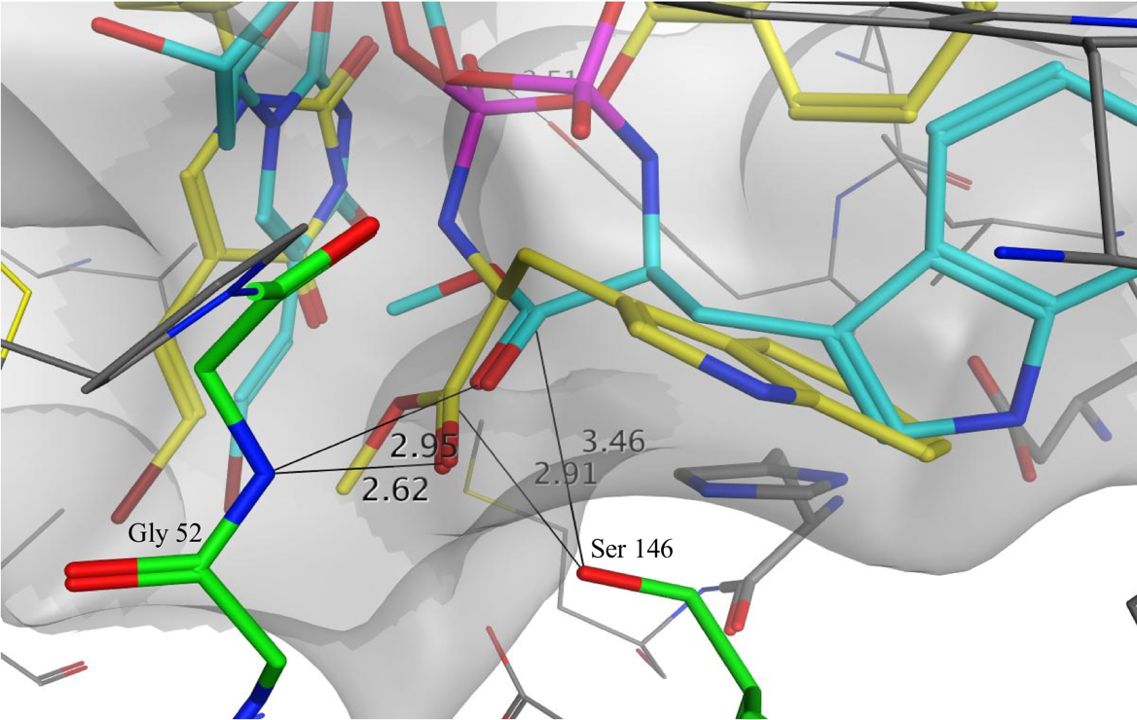


**Figure 4S.** Docking of R_P_ (yellow) and S_P_ (cyan) diastereomers of compound **35** in the catalytic site of carboxypeptidase Y (Cathepsin) showing more favourable interactions of the R_P_ isomer over the S_P_ isomer.

The docking of the diastereoisomers of **35** showed a more favourable interaction between **R**_P_ diastereoisomer (yellow) with the active site of the enzyme than the S_P_ diastereoisomer (cyan), Figure 4S. The ester carbonyl C=O group in the case of the R_P_ diastereoisomer is 2.91 A^o^, while the S_P_ counterpart is 3.46 A^o^ away from the side-chain OH group of the nucleophilic catalytic residue Ser 146. Also, the same carbonyl group is hydrogen bonded to the NH backbone group of residue Gly 52 in a better way in the case of the R_P_ diastereoisomer (2.62 A^o^) compared to the S_P_ diastereoisomer (2.95 A^o^). The docking study suggests that faster metabolised diastereoisomer (**35**s) might have the R_P_ configuration.

1. Corresponding author.

   e-mail address: [kandils1@cardiff.ac.uk](mailto:kandils1@cardiff.ac.uk) (S. Kandil) [↑](#footnote-ref-1)
